# Catalpol ameliorates CFA-induced inflammatory pain by targeting spinal cord and peripheral inflammation

**DOI:** 10.3389/fphar.2022.1010483

**Published:** 2022-10-24

**Authors:** Baoxia Zhao, Jie Fu, Huadong Ni, Longsheng Xu, Chengfei Xu, Qiuli He, Chaobo Ni, Yahui Wang, Jiao Kuang, Mengjie Tang, Qiyang Shou, Ming Yao

**Affiliations:** ^1^ School of Pharmaceutical Sciences, Zhejiang Chinese Medical University, Hangzhou, China.; ^2^ Department of Anesthesiology and Pain Research Center, The First Hospital of Jiaxing Or The Affiliated Hospital of Jiaxing University, Jiaxing, China

**Keywords:** catalpol, inflammatory pain, HDAC4/PPAR-γ -signaling pathway, NF-κB/NLRP3 inflammatory axis, astrocyte activation, peripheral pain

## Abstract

Chronic, inflammatory pain is an international health concern that severely diminishes individuals’ quality of life. Catalpol is an iridoid glycoside derived from the roots of Rehmannia glutinosa that possesses anti-inflammatory, antioxidant, and neuroprotective properties for the treating multiple kinds of disorders. Nevertheless, catalpol’s impacts on inflammatory pain and its potential methods of action are still unclear. The purpose of this investigation is to determine the mechanism of catalpol to reduce the inflammatory pain behaviors in a rat model with complete Freund’s adjuvant (CFA). Catwalk, Von-Frey, and open field testing were performed for behavioral assessment. Western blot analysis and real-time quantitative PCR (RT-PCR) were employed to identify variations in molecular expression, while immunofluorescence was utilized to identify cellular localization. Catalpol effectively reduced CFA-induced mechanical allodynia and thermal hyperalgesia when injected intrathecally. Moreover, catalpol can regulate the HDAC4/PPAR-γ-signaling pathway in CFA rat spinal cord neurons. Meanwhile catalpol significantly decreased the expression of the NF-κB/NLRP3 inflammatory axis in the spinal cord of CFA rats. In addition, both *in vivo* and *in vitro* research revealed that catalpol treatment inhibited astrocyte activation and increase inflammatory factor expression. Interestingly, we also found that catalpol could alleviate peripheral pain by inhibiting tissue inflammation. Taken together, the findings declared that catalpol may inhibit inflammatory pain in CFA rats by targeting spinal cord and peripheral inflammation.

## 1 Introduction

Inflammatory pain is among the highest prevalent kinds of chronic pain detected clinically and is mainly triggered *via* injury and subsequent inflammation of peripheral tissues (Li et al., 2021). It is characterized by persistent, spontaneous pain and hyperalgesia (Descalzi et al., 2015; Baral et al., 2019). Inflammatory pain adversely influences patients’ quality of life, moreover it imposes a major financial penalty (Gereau et al., 2014). Common analgesics are the standard medication for inflammatory pain, which established a reasonable level of success. However, the incidence of severe adverse events reduces the efficacy of the medication (Carter et al., 2014; Eccleston et al., 2017). Consequently, new reliable and innovative anti-inflammatory painkillers are required.

Histone deacetylases (HDACs) limit gene transcription through deacetylating histones. The genome of mammals has at minimum 18 HDAC genes that encode four classes of HDAC proteins: class I (HDAC1, 2, 3, and 8), class II (HDAC4, 5, 7, 9 in IIa, and HDAC6, 10 in IIb), class III (sirtuin1-7), and class VI (HDAC11) (Haberland et al., 2009). These HDAC genes exhibit differential expression in the neurological system (Chauchereau et al., 2004; Ren et al., 2009). Moreover, a proof linking epigenetic control of genes to chronic pain is emerging (Hou et al., 2018; Sanna and Galeotti, 2018). A previous research showed that class IIa HDACs were found to exhibit upregulation in response to complete Freund’s adjuvant (CFA), and intrathecal injection of class IIa histone deacetylase (HDAC) inhibitors inhibited complete Freund adjuvant-induced hyperalgesic inflammation, deleted the functional domain of HDAC4, and increased the latency in response to nociceptive stimuli suggesting that HDAC4 is a nociceptive enhancement of and key contributor to spinal mechanisms (Bai et al., 2010; Crow et al., 2015). Yang et al. discovered that peroxisome proliferator-activated receptor γ (PPAR-γ) may be another important pro-survival transcription factor targeted by HDAC4 (Yang et al., 2011). PPAR-γ is a ligand-activated transcription mediator that performs a crucial function in the gene expression regulation. According to a previous research, stimulation of PPAR-γ inhibits inflammatory pain (Morgenweck et al., 2010). In addition, prior investigations have demonstrated that nuclear factor-kappa B (NF-κB) engages in the inflammatory reaction by triggering the nuclear Nod-like receptor protein 3 (NLRP3) inflammasome (He et al., 2016; Chen et al., 2020). As per the research, targeting NF-κB/NLRP3 signaling-mediated neuronal inflammation may be advantageous for managing the chronic agonizing pain pattern (Derangula et al., 2022). In the present work, we assessed the function of HADC4/PPAR-γ in inflammatory pain, and whether catalpol can reduce inflammatory pain through modulating the HDAC4/PPAR-γ signaling pathway and inhibiting the NF-κB/NLRP3 inflammatory axis.

Recently, there is an emerging proof that glial cells are implicated in the development of chronic pain (Chiang et al., 2007; Cao and Zhang, 2008; Rogers and Merrill, 2022; Tang, 2022). Both astrocytes and microglia are stimulated in the spinal cord after peripheral neurological damage and associated inflammation in tissues. Through generating neuronal regulators including growth elements, proinflammatory cytokines, and chemokines, activated glial cells induce and sustain chronic pain (Gao and Ji, 2010; Fang et al., 2022; Zhang et al., 2022). The suppression of spinal glial activity was, consequently, proposed as a possible innovative therapy for persistent inflammatory pain.

Pain sensing is a complicated phenomenon that typically requires the stimulation of local nociceptive neurons (nociceptors) that transmit pain-signaling messages to the spinal cord and brain (Jennings et al., 2022). As an immunological potentiator, CFA enhances cell-mediated immunity and boosts the synthesis of immunoglobulins. The CFA-induced responses result in tissue inflammation and the generation of cytokines at the injection location (Basbaum et al., 2009; Ahmed et al., 2018). Catalpol is a widely distributed iridoid glycoside that is mostly retrieved from the roots of Rehmannia glutinosa and often utilized as conventional therapy in China (He et al., 2021). Catalpol was observed to possess multiple therapeutic properties, including anti-inflammatory, antioxidant, and neurological protective properties towards several illnesses (Zhang et al., 2007; Hu et al., 2010; Wang et al., 2019). It has been established that catalpol can diminish depressive-like behaviors, inhibit inflammatory reaction, and limit abnormal reactive oxygen species (ROS) formation (Wang et al., 2021). This painkiller action may be associated with the suppression of active microglia and cytokines (IL-1β, IL-6, and TNF-a) in addition to the triggering of NF-κB overexpression in the spinal cord (Wang et al., 2021). This indicates that catalpol has therapeutic promise for treating chronic pain. However, it is unknown whether catalpol performs apart as an analgesic in inflammatory pain, and the exact mechanism through which this would occur is similarly obscure. This study aims to reveal how catalpol alleviates inflammatory pain behaviors in a rat model having CFA.

## 2 Materials and methods

### 2.1 Animals

Standard male Sprague Dawley rats- weighted from 200 to 220 g- were acquired through Shanghai Leagene Biotechnology Co. They were placed at a chamber with regulated temperature (22 ± 2°C), relative humidity of 50%–60%, and a 12/12-h light-dark cycling. The rats had freely available supply of food and water in their compartment. At least 1 week was spent acclimating the rats to laboratory settings before any studies were conducted. The Institutional Animal Care and Use Committee of Jiaxing University (Jiaxing, China) and the International Association for the Study of Pain (IASP) authorized the animal testing techniques. Every attempt was considered to limit the animals’ quantity and distress.

### 2.2 Establishment of an inflammatory pain model and drug administration

The rats were injected intraplantarly with 100 μL of CFA into the left hind paw to produce an inflammatory pain model. Catalpol (CAT, Catalog No. HY-N0820, Med Chem Express, United States) was solubilized in saline. GW9662 (PPAR-γ antagonist, Cat. No. HY-16578, Med Chem Express, United States) was solubilized in sterile saline with 5% DMSO (Cat. No. D2650, Sigma -Aldrich, United States) and 20% Tween 80. CFA (F5881) was acquired through Sigma. The specific experimental groupings and catalpol application management are described below.

The experiment was divided into a total of five parts.

1) A total of 96 rats were used to analyze the effect of catalpol on the pain behavior of CFA rats.

**FIGURE 1:**
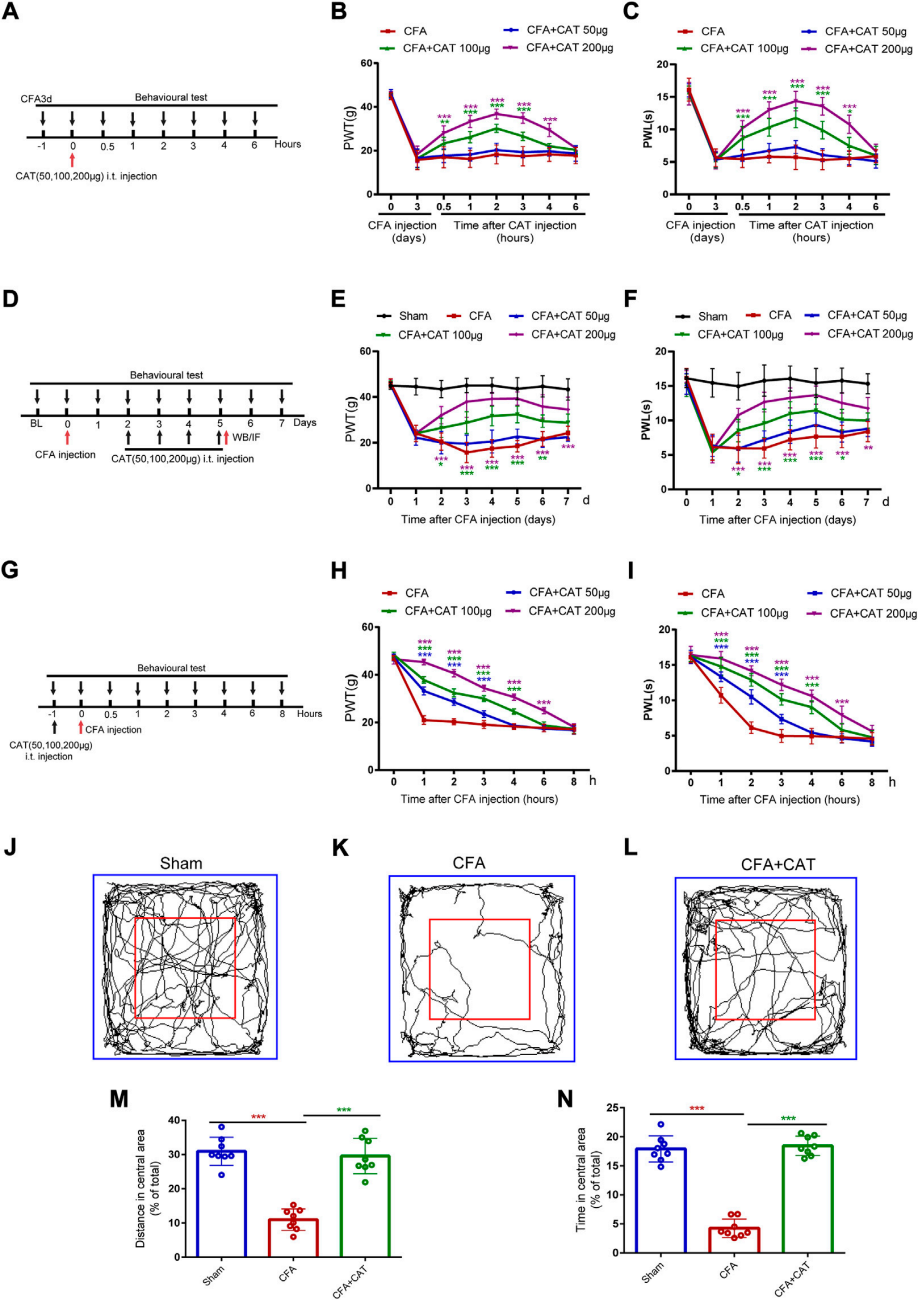
Catalpol could alleviate CFA-induced inflammatory pain in rats. **(A).** Timeline for single treatment of catalpol. **(B,C).** Effect of signal treatment of catalpol (50, 100, 200 μg, i t.) on PWT in response to Von Frey filament stimulation and PWL in response to thermal stimulation. PWT: Paw withdrawal threshold; PWL: Paw withdrawal latency. **(D).** Timeline for repeated injections of catalpol. **(E,F).** Effect of repeated injections of catalpol (50, 100, 200 μg, i. t.) on PWT and PWL. **(G).** Timeline for pre-administered treatment of catalpol. **(H,I).** Effect of pre-administered treatment of catalpol (50, 100, 200 μg, i. t.) on PWT and PWL. (*p < 0.05, **p < 0.01, and ***p < 0.001 vs. the CFA group; n = 6, two-way repeated measures ANOVA). **(J–L).** Representative traces of locomotor activity in the Open field. **(M).** Distance traveled in the central area in the Open field. **(N).** Time spent in the central area in the Open field. (∗∗∗*p* < 0.001 vs. CFA group; n = 8, one-way ANOVA).

To determine whether a single dose of catalpol could alleviate established CFA-induced inflammatory pain, 24 rats were randomly divided into four groups: CFA group, CFA + CAT50μg group, CFA + CAT100μg group, and CFA + CAT200μg group (6 rats in each group). Pain thresholds were measured 1 h before and 0.5, 1, 2, 3, 4, and 6 h after CFA3d single administration (Figure 1A).

To investigate whether repeated intrathecal injections of catalpol could reverse the established inflammatory pain in CFA rats, 24 rats were randomly divided into CFA group, CFA + CAT50μg group, CFA + CAT100μg group and CFA + CAT200μg group (6 rats each). CAT were administered one time every day for 4 days start from second to fifth postoperative days (POD). Behavior assessments were completed daily on CFA1d and 1 h following CAT administration (Figure 1D).

To determine whether early treatment with catalpol inhibited the development of CFA, 24 rats were randomly divided into CFA group, CFA + CAT50μg group, CFA + CAT100μg group, and CFA + CAT200μg group (6 rats in each group). Behavioral tests were performed 1 h before CFA injection and 1, 2, 3, 4, 6 and 8 h after catalpol injection (Figure 1G). 24 rats were randomly divided into Sham group, CFA group, CFA + CAT200μg group on the 4th day after administration of CFA and gait analysis. (*n* = 8).

2) A total of 88 rats were used to study the modulatory effect of catalpol on the HDAC4/PPAR-γ signaling pathway in the spinal cord neurons of CFA rats. To investigate the endogenous expression changes of HDAC4 and PPAR-γ in Sham group and CFA rats, 24 rats were randomly divided into: Sham group, CFA1d group, CFA3d group, and CFA7d group for Western blot assay, 4 rats in each group. Immunofluorescence staining for HDAC4 and PPAR-γ was performed on the 5th postoperative day (n = 4 rats). To further elucidate the role of p-HDAC4 in CFA rats, a total of 18 rats were randomly divided into CFA group, CFA + si-NC group, and CFA + si-HDAC4 group (*n* = 6), and HDAC4 siRNA (5 μg) was given intrathecally once a day from POD2-5 days to measure mechanical and thermal pain threshold changes. At the end of pain measurement, samples were taken for Western blot to detect the expression of p-HDAC4 (*n* = 4). 18 rats were randomly divided into CFA group, CFA + si-HDAC4 group, and CFA + si-HDAC4+GW9662 group (*n* = 6), and HDAC4 siRNA (5 μg) and GW9662 (10 mg/kg) were given intrathecally once a day from POD2-5 days, and pain was measured 2 h after administration (*n* = 6). After the end of pain measurement on the 4th day after administration, the material was taken for Western blot to detect the change of PPAR-γ expression (*n* = 4). A total of 12 rats were randomly divided into Sham group, CFA group and CFA + CAT200μg group, 4 rats in each group, and Western blot was performed to detect the protein expression of p-HDAC4 and PPAR-γ. The fluorescence intensity of p-HDAC4 and PPAR-γ in Sham group, CFA group, and CFA + CAT200μg group was detected by immunofluorescence staining (*n* = 4).
3) To analyze the effect of catalpol on NF-κB/NLRP3-inflammatory axis, Western blot and immunofluorescence were used to detect the protein expression and fluorescence intensity of NLRP3, p-NF-kB in Sham group, CFA group, and CFA + CAT200μg group.
4) To investigate whether the analgesic effect of catalpol was related to the inhibition of astrocyte activation and down-regulation of spinal inflammatory mediators, rats were divided into Sham group, CFA group, and CFA + CAT200μg group, and the protein expression of GFAP, iNOS, IL-1β, and TNF-a in the dorsal horn of rat spinal cord was detected by Western blot (*n* = 4). Immunofluorescence was also used to detect the fluorescence intensity of GFAP in Sham, CFA, and CFA + CAT200μg.
5) A total of 42 rats were used to explore the analgesic effects of catalpol in the periphery. A total of 18 rats were divided into CFA, CFA + CAT 2.5 mg/kg, and CFA + CAT 10 mg/kg (*n* = 6) for single subcutaneous administration on day 3 after CFA injection, and mechanical and thermal pain testing 1 h before and 1, 2, 3, 4, 6, and 8 h after administration (Figure 5A). A total of 12 rats were randomly divided into Sham, CFA3d, and CFA + CAT10 mg/kg taken at 4 h after administration for HE (*n* = 4). Another 12 rats were subjected to Western blot to detect the protein expression of COX-2, IL-1β, and TNF-a in the Sham, CFA3d, and CFA + CAT10 mg/kg groups (*n* = 4).

### 2.3 Intrathecal catheterization

Referring to earlier studies (Ni et al., 2022), rats were thoroughly sedated with pentobarbital (50 mg/kg, i. p.) and afterwards the spinal cord was incised in 2.5-cm length using a sterile and specifically constructed PE-10 tube (Comet Bio, China), which was introduced into the expanded, subarachnoid cavity of the lumbar region. The unfixed point of the PE-10 tube was introduced into the subcutaneous tissue of the back to provide medication. The next day, 10 μL of lidocaine (2%) was administered into the catheter to verify the efficiency of the catheterization. For further examinations, only rats with full paralysis of the two rear legs following lidocaine injection were studied.

### 2.4 Mechanical allodynia

As indicated before, mechanical allodynia was evaluated with a sequence of von Frey monofilaments (BME-404, Institute of Biomedical Sciences, Chinese Academy of Medical Sciences) that were calibrated (Fu et al., 2021a). Prior to conducting the experiment, rats were acclimatized for minimum 30 min in a singular, transparent Plexiglas box (grid: 0.5 cm × 0.5 cm; box: 10 cm × 10 cm × 15 cm) on a metal mesh bottom. We put the monofilament to the plantar side of the rat’s hind paw following 30 min of acclimatization until a favorable reaction (paw withdraw or foot lick) was seen. The paw withdrawal threshold (PWT) was determined by averaging the results of five consecutive experiments. All behavioral analytical techniques were carried out by scientists who protected the confidentiality of the treatment group.

### 2.5 Thermal hyperalgesia test

Rats’ responsiveness to heat stimulation was detected by calculating the paw withdrawal latency (PWL) of their left rear paw. The animals were kept on a heated tray (YLS-6B, Shanghai, China) with a continuous temperature of 52°C. The endpoint was described by removing the paw then retracting or licking it, and the endpoint reaction delay time was obtained. PWL is the period between the beginning of heat and the retraction of the paw. To prevent tissue injury, the maximum latency at the endpoint was set to 20 s. All trials were performed thrice and the mean was calculated.

### 2.6 CATWALK automated gait analysis

Gait testing was conducted employing the CATWALK XT system (Aster Wee Information Technology) to analyze voluntary foot drop and gait patterns in rats. The device comprises a glass surface with an isolated passage that permits the rat to go across one side to the next. The principle is that green light reaches the plate from the long side and is totally reflected onto the glass surface, which has an isolated passage with a red backlight. A camera that can capture photos at extremely fast speeds was situated below the device captures photos of every paw-illuminated region and transfers the results to gait analyzing programs (CATWALK XT, Aster Wee Information Technology) as the rat travel down the hallway and touch the glass surface with their paws. Coordinated and intensified data without area data, were demonstrated in the experiment, related to inflammatory pain intensity in the CFA model and were sensitive to analgesic treatment (Xu et al., 2019). Therefore, in this study, we chose the following parameters: 1) Coordination data: Swing and Swing speed. Swing is the length of time spent in the swing phase of walking. Swing speed is the ratio of the stride length to the swing time is the measure of swing velocity during the swing phase. 2) Area data: Print area is the total amount of floor space, measured in cm^2^, that was covered by all paw photos throughout the stance phase. 3) Intensity data: mean intensity; the contacted zone by the hind paw for the entirety of a step cycle is referred to as the mean intensity. We used the left hind paw/right hind paw (LH/RH) equation to exclude the influence of confounding variables when illustrating the variance in intensity and area values for the ipsilateral (left) hind paw.

### 2.7 Open field test

Open field evaluation was utilized to gauge exploratory behavior. In the darkness of the chamber, a rat was put in the middle of a cage (100 cm × 100 cm × 50 cm) following 30 min of habit formation. Then, a recording camera was utilized to capture the rat’s exploratory behaviors for 10 min in an open field. Using Jliang program (Shanghai, China), proportion of length travelled and duration elapsed in the middle zone were gathered as input. To reduce rats smell stimulus between measures, the testing compartment was washed with 10% alcohol prior to every experiment to remove olfactory stimulus, including odor and waste left by the preceding rat.

### 2.8 siRNA transfection

Gene Pharma (Shanghai, China) supplied HDAC4 small interfering RNA (siRNA). Two days to 5 days following CFA administration, rats were transfected through intrathecal infusion of HDAC4 siRNA (5 µg/20 µL) one time every day for 4 days in a row. The sense strand of HDAC4 siRNA has the sequence 5′- GGA​TGA​GCC​CTA​CCT​AGA​T-3'. For the efficacy verification of HDAC4 expression suppression, Western blots were performed on the L4-L6 spinal cord parts.

### 2.9 Rat primary astrocyte cultures

Primary astrocytes were cultivated as stated before (Ryu et al., 2019). The cerebral cortex of postnatal male Sprague Dawley rats was used to recover cultures of primary glial cells. The cerebral cortex of rats was removed in sterilized, cold PBS, fragmented into single cells using 0.125% trypsin at 37°C, and then underwent a filteration through nylon net with 70 mm pores. Two weeks were spent cultivating all cells in high glucose DMEM with 10% FBS and 1% penicillin in 75 cm^2^ flasks. To acquire primary astrocytes, 75T flasks having blended glial cells were covered with aluminum foil and agitated for one night at 120 rpm on a rotary shaker to separate microglia. After incubating the 75T flasks with removal conditioned media, the cells were rinsed thrice using PBS, treated with trypsin, and underwent a centrifugation at 2000 rpm for 30 min. Primary astrocytes were harvested following being centrifuged, then utilized in the research. Based on the immunological reactivity of glial fibrillary acidic protein (GFAP) demonstrating an astrocytic structure with activities coming from the soma, the purity of the produced astrocytes was between 80% and 95%.

### 2.10 CCK8 assay

Using a CCK8 test, the impact of catalpol on the viability of LPS-treated astrocytes was determined. Briefly, the cultures enriched astroglia were planted at a cell concentration of 4 ×10^4^ cells/well in 96-well plates. The cells were then treated for 6 h with varying doses of catalpol (0, 125, 250, 500, and 1000 µM) with or without LPS (1 μg/ml). After the incubation period completion, 10 µL of CCK8 testing reagent (Dojindo, Japan) was applied to every well, followed by an extra 1 h of incubation at 37 °C. At 450 nm, the absorbance (OD) was calculated utilizing a Multiskan GO spectrophotometer (United States).

### 2.11 H&E staining

Skin tissue from the left hind paw was preserved in buffered 4% paraformaldehyde for 24 h, followed by 3–5 h of tap water washing. The cubes of paraffin were sliced to a thickness of 5 μm. In accordance with a previously reported methodology, the slices were degreased, dried, and dyed with hematoxylin and eosin (H&E) stain (Hua et al., 2022). Photos were acquired through use of a light microscope (Olympus BX 51, Japan).

### 2.12 Western blot

The procedure for extracting proteins was similar to an earlier experiment (Fu et al., 2021b). Through i. p. injection of sodium pentobarbital (100 mg/kg), rats were fully sedated. Spinal cord parts from lumbar4 to 6 and skin tissues were harvested. Electrophoresis of 40 μg of protein samples was performed on SDS polyacrylamide gels. After transferring the proteins to the membrane, they were masked with 5% skim milk for 2 h at room temperature, and then primary antibody was incubated with them for one night at 4°C: rabbit anti-HDAC4 antibody (GTX110231, 1:1000), rabbit anti-p-HDAC4 antibody (Ser632, GTX50237, 1:1000), rabbit anti-PPAR-γ antibody (16643-1-AP, Proteintech, 1:1000), rabbit anti-GFAP (12,389, CST, 1:1000), rabbit anti-p-NF-κB p65 antibody (Ser536, AF 2006, affinity, 1:1000), rabbit anti-NLRP3 antibody (DF7438, Affinity, 1: 1000), rabbit anti-iNOS antibody (18985-1-AP, Proteintech, 1:1000),rabbit anti-IL-1β (AF5103, Affinity, 1: 1000), rabbit anti-TNF-a (AF7014, Affinity, 1: 1000), rabbit anti-COX-2 (27308-1-AP, Proteintech, 1:1000) and rabbit anti-GAPDH (AF7021, affinity, 1:2000). After washing the membranes in Tris-buffered saline containing Tween-20, they were placed in an incubation with a solution containing horseradish peroxidase-labeled goat anti-rabbit secondary antibody (Jackson, 1:2000) at room temperature for 2 h. Immunoresponsive bands were identified utilizing a stimulated chemiluminescence assay (Thermo Scientific) and subjected on X-ray film. The results were standardized relative to the GAPDH loading control. Finally, ImageJ software was utilized to quantify the blots.

### 2.13 Real- time quantitative PCR

Tissues (L4-L6 parts of the spinal cord) were treated for homogenization, and total RNA was retrieved utilizing trizol reagent (Takara Bio Inc, Japan). Following this, the isolated RNA was reverse transcribed to cDNA. Samples were prewarmed at 95°C for 30 s and then confined to 40 amplification periods (95 °C for 5 s then 60°C for 30 s) for cDNA formation. IL-1β, TNF-a and β- actin were supplied by Shanghai Sankyo Bioengineering Co. The sequencing of the sense strand of IL-1β was 5′TGT​TTC​CCT​CCC​TGC​CTC​TGA​C-3′, and the sequencing of the antisense strand was 5′-CGA​CAA​TGC​TGC​CTC​GTG​ACC-3'. The sequencing of the sense strand of TNF-a was 5′- AGC​ACG​GAA​AGC​ATG​ATC​CG-3′ and the antisense chain sequencing was 5′-TGA​GAA​GGC​TGA​GGC​ACA-3'. β-actin had a sense chain sequencing of 5′- CAT​CCT​GCG​TCT​GGA​ACC​TGG -3′ and an antisense chain sequencing of 5′- TAA​TGT​CAC​GCA​CGA​TTT​CC -3'. The relevant expression of the genes was measured by applying the 2-ΔCt method.

### 2.14 Immunofluorescence staining

Immunofluorescence labeling was carried out as reported before (Fu et al., 2021b). Subsequently, L4-L6 spinal cord tissue parts were extracted, kept in 4% paraformaldehyde for 24 h at 4°C, and dried in a gradient sucrose mixture (15%–30%) for 48 h at 4°C. Slices were infiltrated with 0.2% Triton X-100 for 15 min and, after that, sealed for 1 h at room temperature with 5% bovine serum albumin. The slices were then incubated for one night at 4°C with various antibodies. p-HDAC4 (Ser632, GTX50237, rabbit 1:100), PPAR-γ (16,643–1-AP, rabbit source, Proteintech, 1: 50), PPAR-γ (Sc-7273, mouse source, Santa, 1: 50), NLRP3 (DF7438, rabbit source, Affinity, 1: 50), p-NF-κB p65 (sc-166748, mouse source, Santa, 1: 100), iba1 (microglia marker, ab48004, goat source, Abcam, 1: 400), NeuN (neuronal marker, ab104224, mouse source, Abcam, 1: 500), and GFAP (astrocyte marker, C9205, mouse source, Sigma-Aldrich, 1: 500). After that, the slices were incubated for 1 h at room temperature with Alexa Fluor-488 (ab150073, donkey anti-rabbit, Abcam, 1: 500), Alexa Fluor-594 (ab150132, donkey anti-goat, Abcam, 1: 500) Alexa Fluor-594 (ab150108, donkey anti-mouse, Abcam, 1: 500) secondary antibodies, and cell nuclei were dyed using 1 μg/ml DAPI (H-1200 VECTAS HIELD anti-fade mounting media including DAPI). Utilizing a multiphoton confocal microscope (Leica Microsystems, Wetzlar, Germany) photos were taken. In addition, referring to the previous references (Shen et al., 2014; Xu et al., 2014; Ni et al., 2022), we performed the comparison of fluorescence intensity between different groups by averaging the fluorescence intensity for the same magnification, the same spinal cord size, and by selecting several regions that did not completely overlap.

### 2.15 Statistical analysis

This experimental data was analyzed with GraphPad Prism (version 6.0, United States) and reported as the mean ± standard deviation (SD). The behavioral data on nociceptive pain were examined employing a two-way, repeated-measures ANOVA coupled with Bonferroni’s post-hoc testing. Utilizing one-way ANOVA accompanied with the student-Newman-Keuls (SNK) post-hoc testing, Western blotting, RT-PCR, and mean immunofluorescence intensity data were contrasted. The statistical significance threshold was a *p* < 0.05.

## 3 Results

### 3.1 Effect of catalpol on pain behaviors in complete freund’s adjuvant rats

#### 3.1.1 Catalpol alleviated complete freund’s adjuvant-induced mechanical allodynia and thermal hyperalgesia

To determine whether a single dosage of catalpol relieves established CFA-induced inflammatory pain, intrathecal injections of catalpol (50, 100, or 200 μg) were conducted on CFA3d. PWT and PWL were carried out 1 h prior to catalpol adminstration, and 0.5, 1, 2, 3, 4, and 6 h following the dosing. Figures 1B,C, show that PWT and PWL increased sharply in the CFA group, which injected with 100 and 200 μg catalpol contrasted with CFA group from 0.5 to approximately 4 h following dosing. (*p < 0.05, **p < 0.01, and ***p < 0.001 vs. the CFA group; n = 6, two-way, repeated-measures ANOVA). However, no significant variations in PWT or PWL were detected following medication with catalpol at a dose of 50 μg. (*p* >0.05 vs. the CFA group; n = 6, two-way, repeated-measures ANOVA).

For determining if recurrent intrathecal infusions of catalpol may counteract preexisting thermal and mechanical hyperalgesia in CFA rats, various dosages (50, 100, and 200 μg) of catalpol were administered one time every day for 4 days start from second to fifth postoperative days (POD). Behavior assessments were completed daily on CFA1d and 1 h following catalpol administration. In comparison with the CFA group, recurrent doses of 100 and 200 μg of catalpol significantly enhanced PWT and PWL in a dose-dependent manner in CFA rats, and the analgesic impact was sustained for 1–2 days following termination of treatment (*p < 0.05, **p < 0.01, and ***p < 0.001 vs. the CFA group; n = 6, two-way, repeated-measures ANOVA, Figures 1E,F). However, catalpol treatment with 50 µg did not alter PWT or PWL irrespective as to whether treatment was recurrent or once (*p* >0.05 vs. the CFA group; n = 6, two-way repeated-measures ANOVA, Figures 1E,F). These data suggest that single and repeated injections of catalpol have potent analgesic effects on CFA rats.

For examining initial treatment with catalpol ability to inhibit the establishment of CFA, catalpol (50, 100, or 200 μg, i. t.) was given 1 h prior to CFA injection. Behavioral analyses were carried out prior catalpol injection, and 1, 2, 3, 4, 6, and 8 h after CFA adminstration. As shown in Figure 1H,I, both PWT and PWL were significantly higher in catalpol (100, 200 μg)-treated CFA rats than in vehicle-treated CFA rats at 1–4 or 6 h. Interestingly, 50 μg of catalpol treatment also altered mechanical and thermal hyperalgesia at 1–3 h (***p < 0.001 vs. the CFA group; *n* = 6, two-way, repeated-measures ANOVA). The current findings declare that catalpol cannot completely limit the development of CFA-induced mechanical and thermal pain but can delay their onset. In conclusion, catalpol can reduce CFA-induced mechanical allodynia and thermal hyperalgesia.

#### 3.1.2 Effects of catalpol on exploratory behavior in complete freund’s adjuvant rats

We then assessed the exploratory behavior of the CFA rats in an Open field testing. The behavioral evaluation was performed on CFA rats after 4 days of catalpol (200 μg, i. t.) treatment. Compared to the sham group, CFA-injected rats saved time and travelled shorter areas in the Open field test’s middle area, which indicated anxiety-like symptoms. Administration of catalpol reversed this effect suggesting that catalpol treatment improved exploratory behavior in CFA rats. (∗∗∗*p* < 0.001 vs. the CFA group; n = 8, one-way ANOVA, Figure 1J–N).

#### 3.1.3 Effects of catalpol on Cat Walk gait parameters in complete freund’s adjuvant rats

**FIGURE 2:**
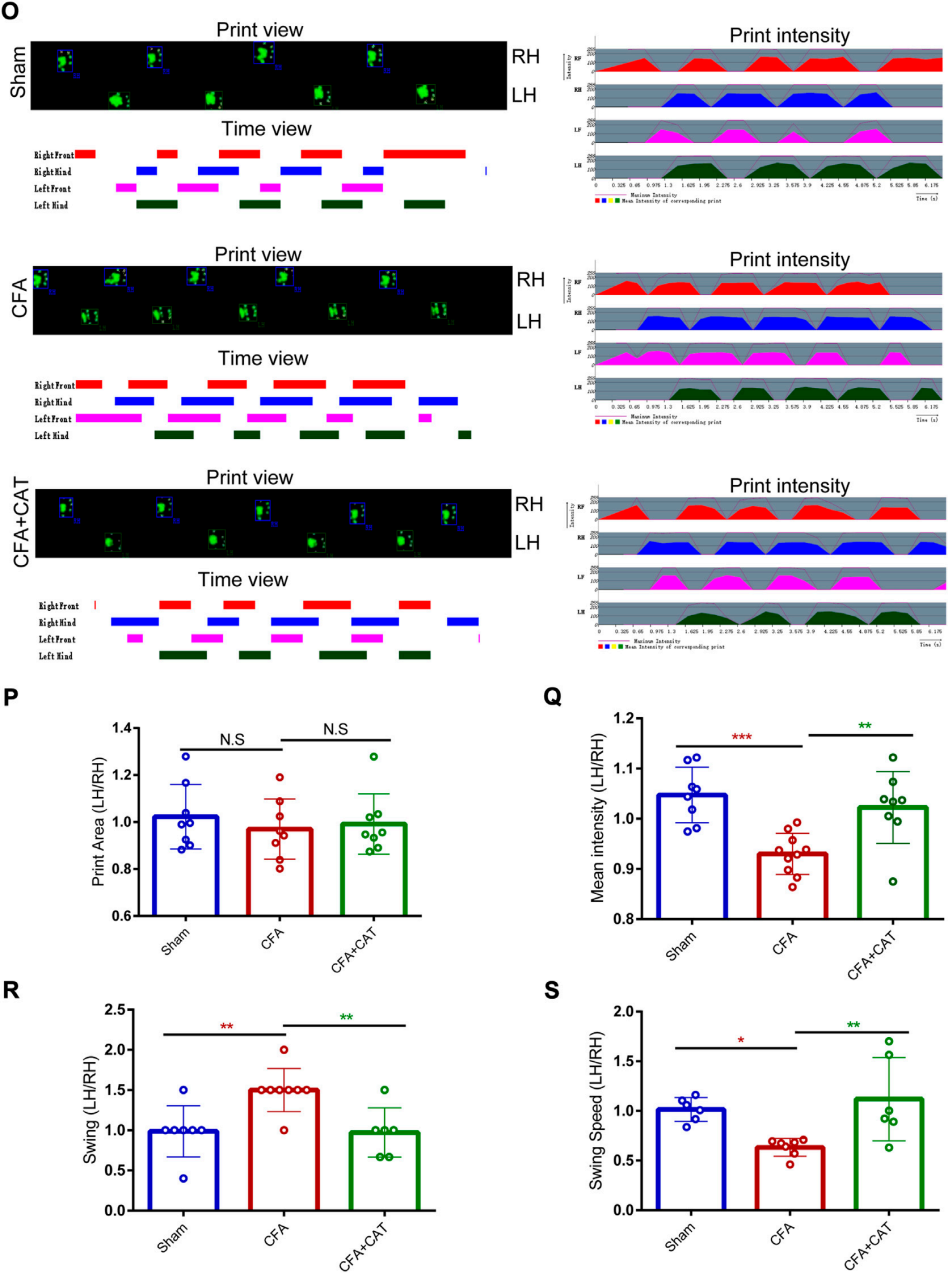
Effects of catalpol on Cat Walk gait parameters in CFA rats. **(O).** Representative CATWALK gait analysis results, including Print view, Timing view and Print intensity. Changes of gait parameters including Print Area **(P)**, Mean intensity **(Q),** Swing **(R),** and Swing Speed **(S)** in Sham, CFA, and CFA + CAT group. (∗*p* < 0.05, **p < 0.01 and ∗∗∗*p* < 0.001 vs. the CFA group; n = 8, one-way ANOVA). All data were calculated as left hind paw/right hind paw (LH/RH) formula to eliminate the effect of confounding factors. LH: left hind paw; RH: right hind paw; N.S. not statistically significant.

CATWALK gait assessment is taken into account as an objective technique to assess chronic pain behavior in inflammatory pain modeling (Heinzel et al., 2020; Hestehave et al., 2020). In the current experiment, we investigated many gait measures, including swing, swing speed, average intensity, and print area, to determine if CFA rats exhibited pain-related behaviors. Cat Walk gait assessment was performed on CFA rats administered catalpol (200 μg i. t.) for 4 days. As demonstrated in Figures 2O–S, gait measures, including mean intensity and swing speed, reduced significantly on Day 5 following CFA injection contrasted with the sham group, although swing duration rose, demonstrating that CFA induced painful sense and locomotor impairment in rats. Nevertheless, medication with catalpol (200 μg) clearly restored the changes in gait characteristics generated by CFA administration in rats. (∗*p* < 0.05, **p < 0.01 and ∗∗∗*p* < 0.001 vs. the CFA group; n = 8, one-way ANOVA). Furthermore, validation images of the paw print area revealed the absence of significant variation between the left hind foot paw print zone of sham rats and that of the CFA and CFA + CAT groups (*p* >0.05 vs. the CFA group; n = 8, one-way ANOVA, Figures 2O,P). These results demonstrate that catalpol is effective in alleviating coordination- and intensity-related pain behaviors in CFA rats.

### 3.2 Catalpol modulates the HDAC4/PPAR-γ-signaling pathway in spinal cord neurons of complete freund’s adjuvant rats

The data presented above suggest that catalpol has a strong analgesic impact on CFA-induced inflammatory pain. To study the potential process of the antinociceptive effect generated by catalpol in CFA rats, we assessed the impact of this therapy on the expression of HDAC4 and PPAR-γ in the spinal cord of CFA rats.

**FIGURE 3:**
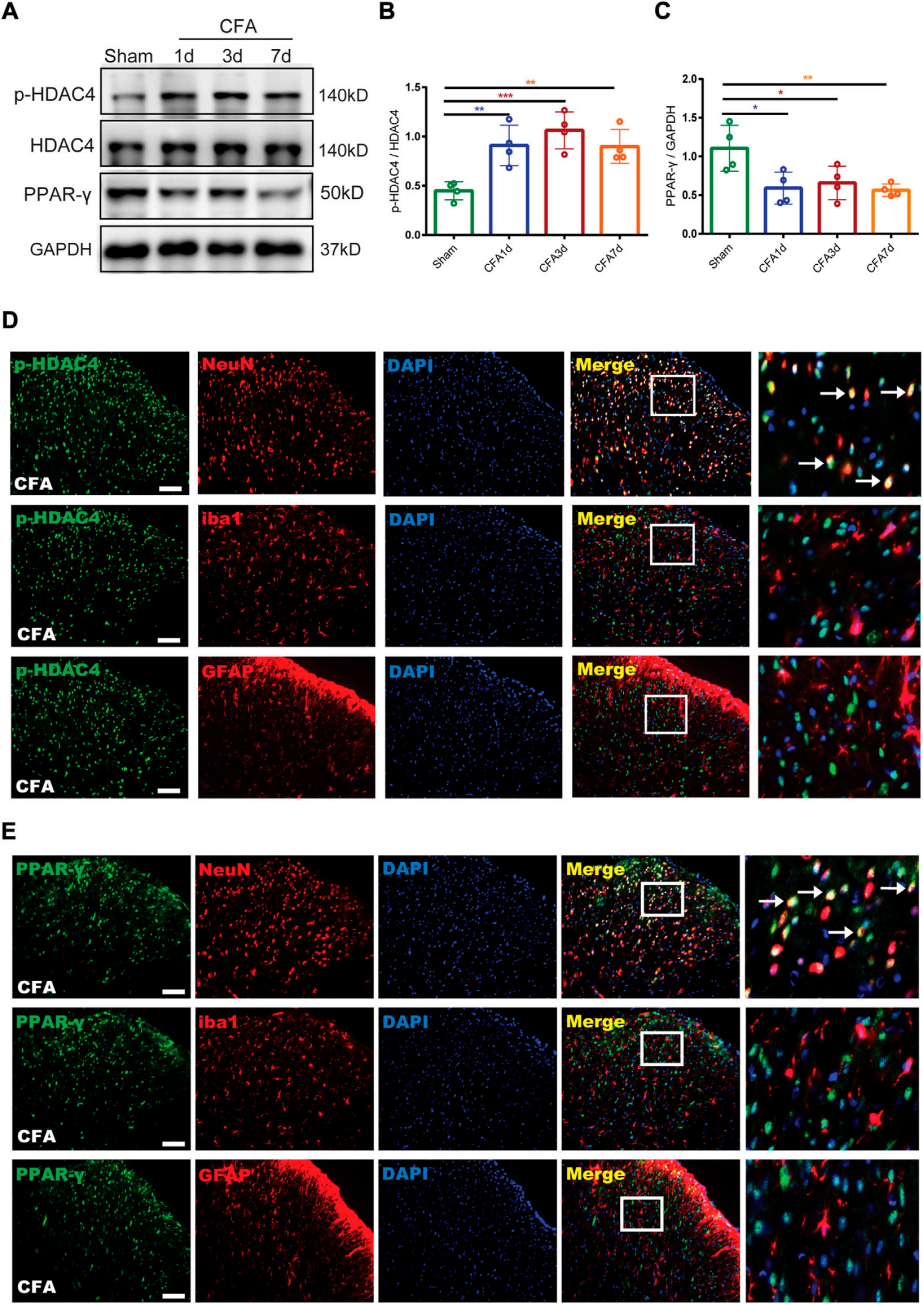
The endogenous expression and cellular localization of p-HDAC4 and PPAR-γ in the spinal cord of CFA rats. **(A–C).** The protein expressions of p-HDAC4 and PPAR-γ in the spinal cord of rats were detected by Western blotting. **(D,E).** The colocalization of p-HDAC4 and PPAR-γ with NeuN, iba1, and GFAP were detected by a double-label immunofluorescence assay in CFA rats. The white arrows indicate colocalization of p-HDAC4 and PPAR-γ with NeuN. Scale bar = 50 μm.

For detecting the endogenous expression and cellular localization of p-HDAC4 and PPAR-γ in the spinal cord, the lumbar spinal cords of rats (L4-L6) were collected 1, 3, and 7 days following the complete development of the CFA model and analyzed by Western blotting and immunofluorescence. Western blotting results showed a significant increase in p-HDAC4 levels on Days 1, 3, and 7 after CFA infusion contrasted with rats in the sham group whereas total HDAC4 expression was unchanged and, in contrast, PPAR-γ expression was reduced (∗*p* < 0.05, **p < 0.01 and ∗∗∗*p* < 0.001 vs. the sham group; n = 4, one-way ANOVA, Figures 3A–C). For determining the cellular localization of p-HDAC4 and PPAR-γ in the spinal dorsal horn, we co-stained p-HDAC4, PPAR-γ with neuronal or glial markers. (NeuN, iba1, and GFAP). The findings declared that p-HDAC4 and PPAR-γ colocalized mainly with neurons, while not with microglia and astrocytes in CFA rats (Figures 3D,E).

**FIGURE 4:**
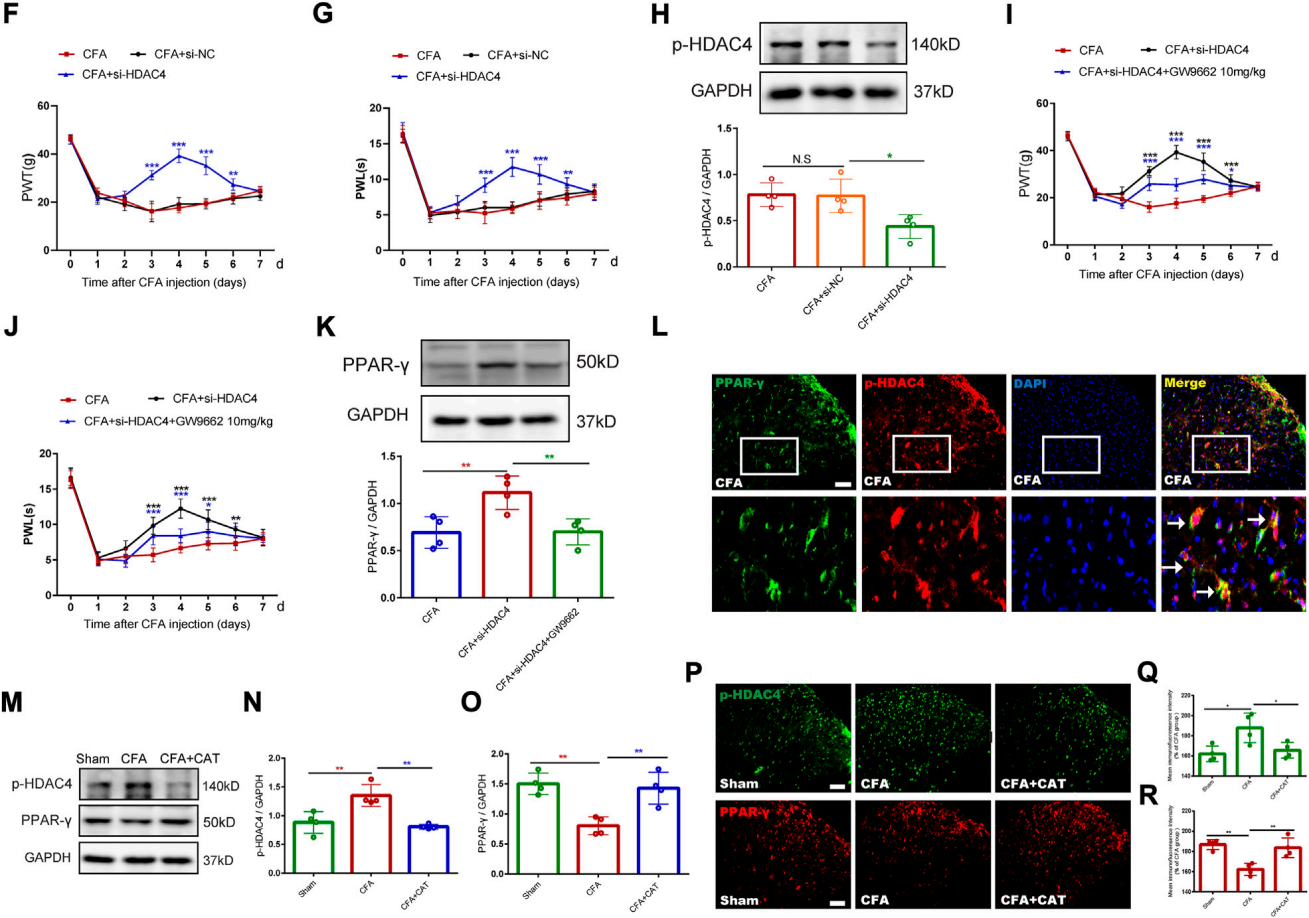
Effect of catalpol on HDAC4/PPAR-γ-signaling pathway in spinal cord of CFA rats. **(F,G).** Effect of intrathecal injection of HDAC4 siRNA (5 μg) on PWT and PWL. (**p < 0.01, and ***p < 0.001 vs. CFA group; n = 6, two-way repeated measures ANOVA). **(H).** The relative protein expression of p-HDAC4. (∗*p* < 0.05 vs. the CFA group; n = 4, one-way ANOVA, N.S: not statistically significant). **(I,J).** Effect of GW9662 administration on PWT and PWL. (*p < 0.05, **p < 0.01, and ***p < 0.001 vs. the CFA + si-HDAC4 group; n = 6, two-way repeated measures ANOVA). **(K).** The relative protein expression of PPAR-γ. (**p < 0.01, vs. the CFA + si-HDAC4 group; n = 4, one-way ANOVA). **(L).** The co-expression of p-HDAC4 and PPAR-γ in the spinal cord of rats was detected by a double-label immunofluorescence assay. The white arrows indicate colocalization of p-HDAC4 with PPAR-γ. Scale bar = 50 μm. **(M-O).** The relative protein expression of p-HDAC4 and PPAR-γ. (**p < 0.01, vs. the CFA group; n = 4, one-way ANOVA). **(P-R).** The fluorescence intensity of p-HDAC4 and PPAR-γ. (*p < 0.05, **p < 0.01, vs. the CFA group; n = 4, one-way ANOVA). Scale bar = 50 μm.

For additional elucidation of the impact of p-HDAC4 and PPAR-γ in CFA rats, HDAC4 siRNA (5 μg) and GW9662 (an antagonist of PPAR-γ) were administered once every day for 4 successive days starting from postoperative Days 2–5 (POD). The results showed that HDAC4 siRNA (5 μg) significantly inhibited the developed mechanical and thermal pain in CFA rats (**p < 0.01, and ***p < 0.001 vs. CFA group; n = 6, two-way, repeated-measures ANOVA, Figures 4F,G). The findings of the following molecular trials declared that repeated intrathecal HDAC4 siRNA administration reduced the expression of p-HDAC4 (∗*p* < 0.05 vs. the CFA group; *n* = 4, one-way ANOVA, Figure 4H). In addition, repeated intrathecal HDAC4 siRNA injections also significantly elevated PPAR-γ expression compared with the CFA group while GW9662 treatment reversed this effect (**p < 0.01, vs. CFA + si-HDAC4 group; n = 4, one-way ANOVA, Figure 4K). Meanwhile, the behavioral results also demonstrated that GW9662 administration partially reduced the analgesic impact of HDAC4 siRNA (*p < 0.05, **p < 0.01, and ***p < 0.001 vs. the CFA + si-HDAC4 group; *n* = 6, two-way, repeated-measures ANOVA, Figures 4I,J). In addition, as shown in Figure 4L **,** the immunofluorescence double-labeling results revealed that p-HDAC4 and PPAR-γ were co-localized. These results suggest that the HDAC4/PPAR-γ-signaling pathway takes a part in CFA-induced inflammatory pain.

Finally, this work evaluated the action of catalpol on the CFA-induced, HDAC4/PPAR-γ-signaling pathway in the rats’ spinal cord. Figures 4M–O, depicts that western blot outcomes declared that catalpol therapy reduced the expression of p-HDAC4, while elevated the expression of PPAR-γ, contrasted with the CFA group (**p < 0.01, vs. the CFA group; n = 4, one-way ANOVA). Immunofluorescence data was similar to the Western blot results (*p < 0.05, **p < 0.01, vs. the CFA group; *n* = 4, one-way ANOVA, Figures 4P–R). The present findings suggest that catalpol modulates the HDAC4/PPAR-γ- signaling pathway in the rats’ spinal cord neurons.

### 3.3 Effect of catalpol on the NF-κB/NLRP3-inflammatory axis in the spinal cords of complete freund’s adjuvant-treated rats

**FIGURE 5:**
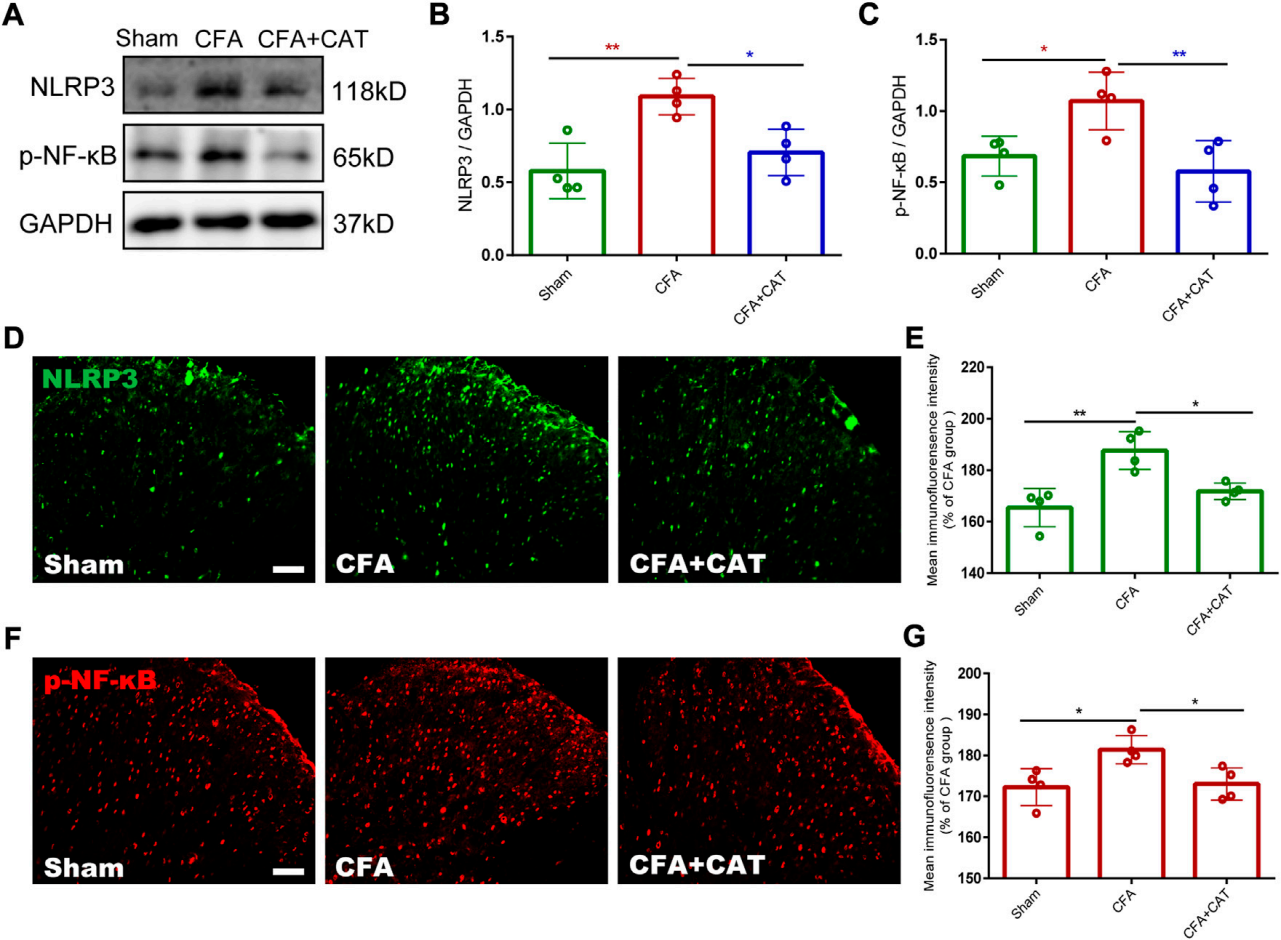
Effect of Catalpol on expression of NF-κB/NLRP3-inflammatory axis in the spinal cord of CFA rats. **(A–C).** The protein expressions of p-NF-κB and NLRP3 in the spinal cord of rats were detected by Western blotting. (*p < 0.05, **p < 0.01, vs. the CFA group; n = 4, one-way ANOVA). **(D,E).** The fluorescence intensity of NLRP3. **(F,G).** The fluorescence intensity of p-NF-κB. (*p < 0.05, **p < 0.01, vs. the CFA group; n = 4, one-way ANOVA). Scale bar = 50 μm.

Previous studies suggest that the NF-κB/NLRP3 inflammatory axis performs a crucial part in pain (Fu et al., 2021b). Therefore, we decided to elucidate the potential molecular mechanisms by which catalpol inhibit inflammatory pain. Subsequently, we assessed the influences of catalpol on the NF-κB/NLRP3-inflammatory axis after CFA injection. Western blot analysis findings demonstrated that the expression of p-NF-κB and NLRP3 in the spinal cord of CFA rats was significantly increased contrasted with the sham group. In contrast, treatment with catalpol reversed this alteration (*p < 0.05, **p < 0.01, vs. the CFA group; *n* = 4, one-way ANOVA, Figures 5A–C). This was confirmed by the subsequent immunofluorescence examination results (*p < 0.05, **p < 0.01, vs. the CFA group; n = 4, one-way ANOVA, Figures 5D–G). The present findings indicated that catalpol can decrease the expression of the NF-κB/NLRP3-inflammatory axis in CFA rats.

### 3.4 Catalpol treatment inhibits the activation of astrocytes and the release of inflammatory factors *in vivo* and *in vitro*

**FIGURE 6:**
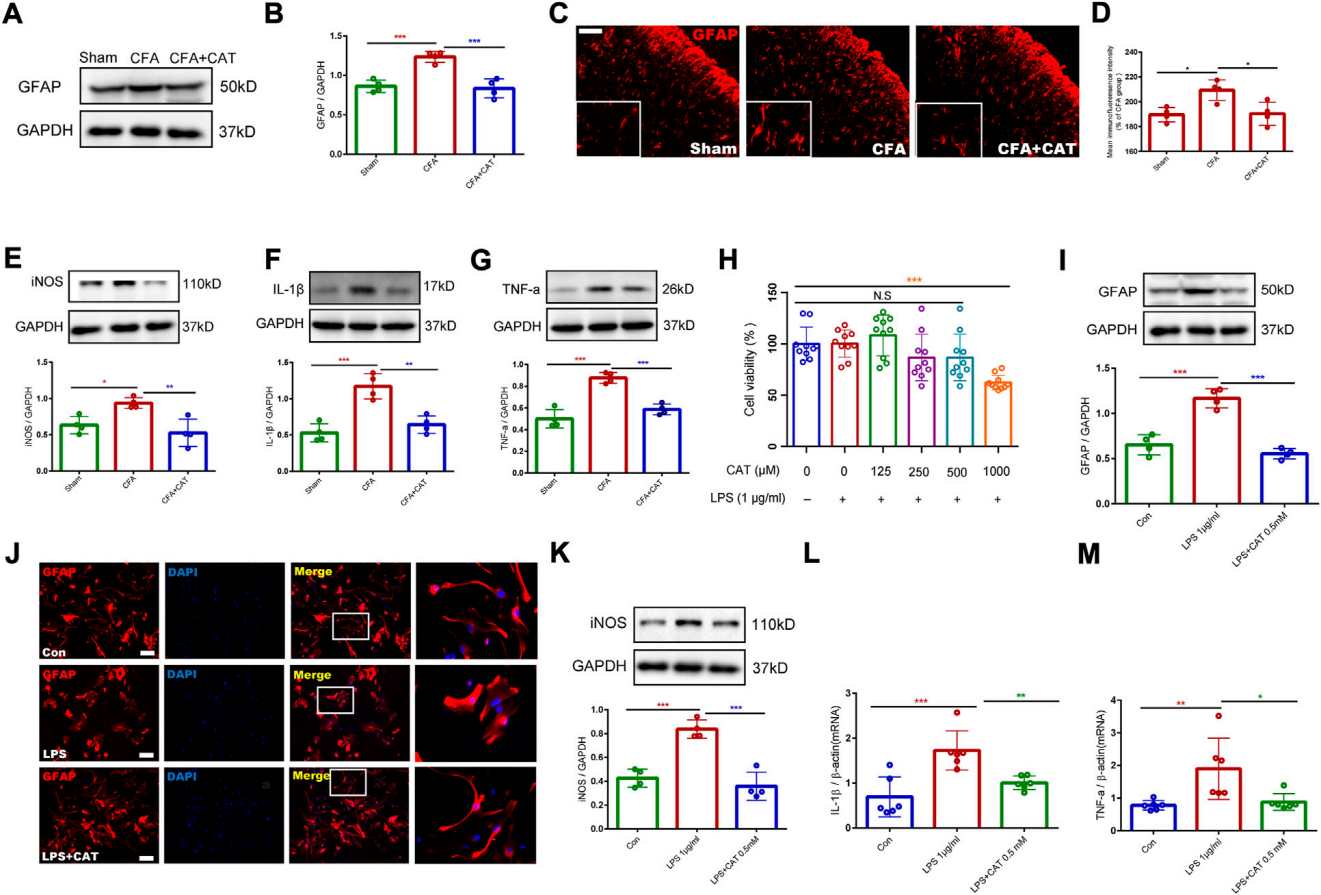
Catalpol treatment inhibits the activation of astrocytes and the release of inflammatory factors *in vivo* and *in vitro*. **(A,B).** The protein expression of GFAP in the spinal cord of rats were detected by Western blotting. **(C,D).** The fluorescence intensity of GFAP in the spinal cord. (*p < 0.05 vs. the CFA group; n = 4, one-way ANOVA). Scale bar = 50 μm. The relative protein expression of iNOS **(E)**, IL-1β **(F),** and TNF-a **(G)** in the spinal cord. (*p < 0.05, **p < 0.01, ***p < 0.001, vs. the CFA group; n = 4, one-way ANOVA). **(H).** Cell viability was measured using CCK8 assay. (***p < 0.001 vs. control; one-way ANOVA). **(I).** The protein expression of GFAP in primary astrocytes. (***p < 0.001, vs. LPS (1 μg/ml) group; one-way ANOVA). **(J).** Immunofluorescence results of GFAP in primary astrocytes. Scale bar = 50 μm. **(K).** The protein expression of iNOS in primary astrocytes. **(L,M).** The mRNA expression of IL-1β and TNF-α in primary astrocytes. (*p < 0.05, **p < 0.01, ***p < 0.001, vs. LPS (1 μg/ml) group; one-way ANOVA).

For further assessing the antinociceptive impact of catalpol correlation with the inhibition of astrocyte stimulation and the decreased expression of inflammatory factors in the spinal cord, the relative protein expression was detected using Western blot testing. The outcomes revealed that GFAP, iNOS, IL-1β, and TNF-a were significantly increased in the CFA group related to the sham group whereas catalpol therapy suppressed this outcome (*p < 0.05, **p < 0.01, ***p < 0.001, vs. the CFA group; n = 4, one-way ANOVA, Figures 6A–G). The findings of immunofluorescence revealed that astrocytes in the CFA group exhibited stronger immunofluorescence intensity and hypertrophic processes than those in the sham group, while catalpol treatment caused suppression of this change. (*p < 0.05 vs. the CFA group; n = 4, one-way ANOVA, Figures 6C,D). These results suggest that catalpol treatment suppresses CFA-induced action of astrocytes and the expression of proinflammatory factors in the spinal cord of rats.

For additional investigation of the impact of catalpol on astrocyte activation and the expression of inflammatory factors, we cultured primary astrocytes and incubated them with LPS (1 μg/ml) for 6 h. We first investigated the influence of catalpol on the viability of LPS-treated, primary-cultured astrocytes. Medication using catalpol (0–1000 μM), subsequent to activation with LPS (1 μg/ml) for 6 h showed that catalpol was toxic to the cells at 1000 μM (***p < 0.001 vs. control; one-way ANOVA, Figure 6H). Consequently, the dosage of catalpol was chosen to be 500 µM in subsequent experiments. Western blot data declared that LPS treatment enhanced GFAP expression related to the control, and this action was limited by pretreatment with catalpol at a dose of 500 μM for 1 h (***p < 0.001, vs. LPS (1 μg/ml) group; one-way ANOVA, Figure 6I). As shown in Figure 6J, the immunofluorescence results also confirmed that astrocytes in the LPS-treated group experienced significant activation, mainly in the form of cytosolic hypertrophy, contrasted with the control group, which was inhibited by catalpol using. Furthermore, catalpol significantly limited LPS-induced expression of inflammatory factors (iNOS, IL-1β and TNF-α) in primary astrocytes (*p < 0.05, **p < 0.01, ***p < 0.001, vs. LPS (1 μg/ml) group; one-way ANOVA, Figure 6K–M). In conclusion, the *in vivo* and *in vitro* data declare that catalpol inhibits the activation of astrocytes and the subsequent release of inflammatory factors.

### 3.5 Catalpol reduces peripheral pain by inhibiting tissue inflammation

**FIGURE 7:**
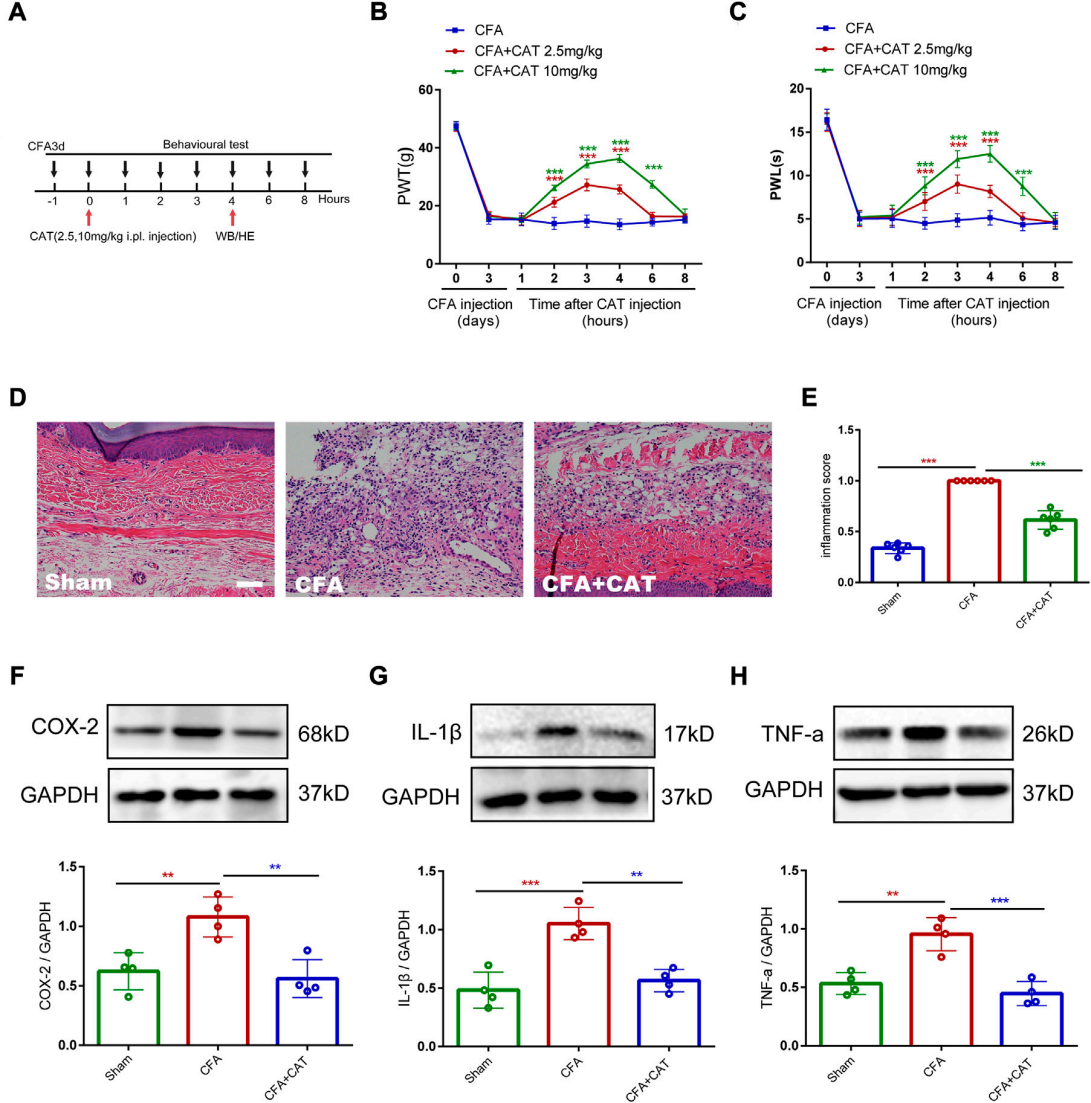
Catalpol reduces peripheral pain by inhibiting tissue inflammation. **(A).** Timeline for single subcutaneous injections of catalpol. **(B,C).** Effect of single subcutaneous injections of Catalpol (2.5 mg/kg and 10 mg/kg) on PWT and PWL. (***p < 0.001 vs. the CFA group; n = 6, two-way repeated measures ANOVA). **(D,E).** H&E staining of paw skin in different groups. (∗∗∗*p* < 0.001 vs. the CFA group; n = 4, one-way ANOVA). Scale bar = 50 μm. **(F–H).** The protein expression of COX-2, IL-1β and TNF-a in the paw skin of rats were detected by Western blotting. (**p < 0.01 and ∗∗∗*p* < 0.001 vs. the CFA group; n = 4, one-way ANOVA).

To explore the analgesic and anti-inflammatory properties of catalpol on the peripherals, the experiment was undertaken on day 3 following CFA administration. Single, subcutaneous injections of catalpol (2.5 mg/kg and 10 mg/kg) significantly limited the mechanical allodynia and thermal hyperalgesia in dose-dependent manners. In addition, the analgesic action of catalpol persisted for 6 h (***p < 0.001 vs. the CFA group; n = 6, two-way, repeated-measures ANOVA, Figures 7B,C). Furthermore, skin samples were taken 4 h following catalpol therapy for Western blot analysis and H&E labeling. H&E labeling indicated that rats in the CFA group possessed abundant lymphocytes and sparse neutrophils compared to control rats whereas catalpol treatment suppressed this effect (***p < 0.001 vs. the CFA group; n = 4, one-way ANOVA, Figures 7D,E). As shown in Figures 7F–H, Western blot outcomes demonstrated that CFA triggered a significant elevation in proinflammatory factors (COX-2, IL-1β and TNF-a), and treatment with catalpol (10 mg/kg) reversed this result (**p < 0.01 and ***p < 0.001 vs. the CFA group; n = 4, one-way ANOVA). The results suggest that catalpol can also reduce peripheral pain by inhibiting tissue inflammation.

## 4 Discussion

To recapitulate, chronic, inflammation-related pain is a worldwide health concern. Currently, inflammatory pain affecting the quality of life of individuals in a significant way in light of the drawbacks of conventional analgesics regarding to efficacy and safety (Cooper et al., 2017). In this work, the analgesic efficacy of catalpol on CFA-induced inflammatory pain was studied, as well as the probable pathways behind this action. Our findings revealed that medication with catalpol reduced the mechanical allodynia and thermal hyperalgesia caused by CFA. Catalpol regulated the HDAC4/PPAR-γ-signaling pathway in CFA rat spinal cord neurons and prevented the stimulation of the NF-κB/NLRP3 inflammatory axis. Studies conducted *in vitro* and *in vivo* demonstrated that catalpol reduced both astrocyte stimulation and the expression of inflammatory factors. Additionally, catalpol relieved peripheral pain by reducing tissue inflammation. These findings give a proof for the existence of novel potential therapy and control options for inflammatory pain.

Earlier research has revealed that repeated medication using catalpol reduces mechanical pain in CCI and SNL neuropathic pain models (Wang et al., 2014). Consistent with this report, our current study shows that both pretreatment and posttreatment with catalpol dose-dependently alleviated CFA-induced mechanical and thermal hyperalgesia. The present findings demonstrate that a single intrathecal infusion of catalpol temporarily reduces pain hypersensitivity generated by CFA while repeated administration shows prolonged analgesic effects. Our findings also suggest that pre-administration of catalpol does not completely prevent the onset of CFA-induced mechanical and thermal hyperalgesia, however, can delay their onset.

In the present study, although we found that early single injections and pre-administration of catalpol temporarily reduced pain hypersensitivity and delayed the development of CFA, respectively, they did not completely prevent CFA-induced mechanical and thermal pain. We were surprised to find that catalpol completely reversed the established mechanical and thermal pain of CFA after repeated intrathecal injections, suggesting that repeated intrathecal injections of catalpol could exert significant analgesic effects. Therefore, in this study we focused on the effect of repeated intrathecal injections catalpol on the established pain behavior of CFA. In addition, previous studies have shown that chronic pain has the potential to increase the emergence of anxiety and produce symptoms of co-morbid anxiety in pain (Shao et al., 2021; Farzinpour et al., 2022; Spinieli et al., 2022), so we investigated whether established persistent inflammatory pain induced by CFA can promote anxiety-like behavior in rats and whether catalpol alleviates after repeated treatment. This was confirmed by our results. We found that CFA-injected rats saved more time and travelled shorter routes in the central area of the Open-field test suggesting anxiety-like symptoms. Administration of catalpol prevented these changes, which suggested that catalpol treatment improves exploratory behavior in CFA rats. As per past research catalpol reduces depressive-like behaviors in CUMS mice *via* oxidative, stress-mediated NLRP3 inflammasome stimulation and neuroinflammation (Wang et al., 2021). This is consistent with our results.

CATWALK gait evaluation was conducted to accurately evaluate mechanical allodynia in persistent pain models, as described in research on inflammatory and neuropathic pain models (Gabriel et al., 2007; Muramatsu et al., 2014). In our investigation, we evaluated numerous gait metrics; like swing, swing speed, mean intensity, and print area, to show pain-related behaviors in CFA rats. The results showed that after CFA injection, gait metrics, like mean intensity and swing speed, decreased significantly, but swing time increased indicating that CFA triggered sensory and motor dysfunction in rats experiencing pain. Even though, catalpol therapy significantly reversed the changes in gait metrics induced by CFA administration in rats. In addition, validation photos of the paw print area exhibited no significant difference between the left hindfoot paw print area of control rats and that of the CFA and CFA + CAT groups. This is aligned with the observations of Xu et al., who discovered that coordination and intensity information, excluding area information, were linked to the degree of inflammatory pain and susceptible to analgesic therapy in the CFA model (Shepherd and Mohapatra, 2018; Xu et al., 2019).

Multiple pathways involving HDAC4 may impact sensory neuron growth, regeneration, and pain. Wen et al. discovered that sciatic nerve damage caused by prolonged constriction enhanced HDAC4 expression in the DRG. This indicated that HDAC4 in the DRG contributed to the neuropathic pain triggered by CCI (Wen et al., 2019). Lai et al. observed that laboratory animals displaying behavioral nociceptive hypersensitivity were experiencing HDAC4 phosphorylation and cytoplasmic redistribution in dorsal horn neurons by administering CFA into the rats’ hind paw. This pilot research conclude that the emergence or persistence of inflammatory pain includes HDAC4-dependent, epigenetic pathways in the spine (Lai et al., 2018). In the present work, we discovered that the phosphorylated HDAC4 expression is enhanced in the spinal cord of CFA rats, while overall HDAC4 expression was unaffected. Moreover, HDAC4 siRNA administration obviously reversed mechanical and thermal hyperalgesia in CFA rats, which is similar to earlier studies. Remarkably, we found that repeated intrathecal injections of HDAC4 siRNA significantly enhanced the expression of PPAR-γ while GW9662 treatment reversed this effect. The behavioral findings declared that GW9662 administration partially reversed the analgesic effect of HDAC4 siRNA. In a previous study, PPAR-γ was found to be an additional pro-survival, transcription factor as a main target of HDAC4 (Yang et al., 2011). This experiment yielded results consistent with those of prior experiments showing that HDAC4 can regulate PPAR-γ. The above results indicate that the HDAC4/PPAR-γ- signaling pathway serves a crucial part in CFA-induced, inflammatory pain. Previous studies have found that catalpol inhibits cardiomyocyte cell death *via* the Neat1/miR-140e5p/HDAC4 axis in diabetic cardiomyopathy. Induction of PPAR-γ by Catalpol mitigated doxorubicin-induced inflammation and oxidative stress in H9C2 cells (Zou et al., 2019; Jiang and Zhang, 2020). Consequently, we examined the influence of catalpol on the HDAC4/PPAR-γ-signaling pathway. The Western blot and immunofluorescence results showed that catalpol treatment reversed the CFA-induced changes in p-HDAC4 and PPAR-γ expression. These results suggest that catalpol could modulate the HDAC4/PPAR-γ-signaling pathway in rat spinal cord neurons.

In addition, previous studies suggest that targeting NF-κB/NLRP3 signaling-mediated neuroinflammation may be beneficial in modulating chronic pain (Derangula et al., 2022). It has been reported that Catalpol protects against spinal cord injury in mice by regulating the MicroRNA-142-mediated HMGB1/TLR4/NF-κB signaling pathway (Xia et al., 2020). Catalpol ameliorates depression-like behavior in CUMS mice through oxidative stress-mediated NLRP3 inflammasome and neuroinflammation (Wang et al., 2021). To further investigate the possible mechanisms involved in the analgesic effect of catalpol in CFA-induced inflammatory pain, we investigated the effect of catalpol on the NF-κB/NLRP3 inflammatory axis. Western blot and immunofluorescence data demonstrated that the expressions of p-NF-κB and NLRP3 were significantly increased in the spinal cords of CFA-treated rats. In contrast, treatment with catalpol blocked this action. The results showed that catalpol can inhibit the expression of NF-κB/NLRP3-inflammatory axis.

In the present study we first found that the HDAC4/PPAR-γ signaling pathway in rat spinal cord neurons is involved in CFA-induced inflammatory pain. However, astrocyte-mediated neuroinflammatory responses also play an important role in chronic pain (Chiang et al., 2007; Kiguchi et al., 2012; Old and Malcangio, 2012). Previous studies have shown that catalpol has anti-inflammatory effects, and Chen et al. found that catalpol was able to inhibit IL-4, IL-5, IL5Rα, and immunoglobulin E (IgE) to alleviate airway inflammation in ovalbumin-induced asthmatic mice (Chen et al., 2017). Therefore, we also further investigated whether catalpol could inhibit the astroglial-mediated spinal neuroinflammatory to attenuate central sensitization. In addition, we simulated CFA-induced neuroinflammation *in vitro* by co-incubating LPS (1 ug/mL) with glial cells, following the study of Hui et al. We were surprised to find that the inflammatory environment provided by LPS during *in vitro* incubation stimulated astrocyte activation and release of inflammatory factors, suggesting a similar pathological process to CFA-induced inflammation *in vivo*. Interestingly, our *in vitro* and *in vivo* results showed that catalpol could inhibit the activation of astrocytes and the expression of released inflammatory factors such as iNOS, IL-1β and TNF-a. In summary, catalpol not only regulates the HDAC4/PPAR-γ signaling pathway in spinal cord neurons, but also inhibits astrocyte-mediated spinal cord neuroinflammation. There is a juxtaposition between the spinal astrocyte cell-mediated inflammatory response and neuronal injury, which together lead to central sensitization and thus participate in CFA-induced inflammatory pain.

Although central sensitization at the spinal cord level plays an important role in the development and maintenance of chronic pain (Santoni et al., 2022), peripheral mechanisms are also clearly involved. After tissue injury, local macrophages generate an inflammatory response and various inflammatory mediators act synergistically to induce and maintain the development of pain hypersensitivity (Hui et al., 2013). Therefore, we further explored the possible peripheral mechanisms involved in the analgesic effect of catalpol. We found that CFA injection increased the expression of TNF-a, IL-1β, and COX-2 in skin tissue, while catalpol plantar injection decreased their expression in paw skin and inhibited mechanical and thermal pain. Catalpol has been shown to have anti-inflammatory effects in previous studies (Bi et al., 2020; Shu et al., 2021), and our results confirm this effect. This suggests that the peripheral analgesic effect of catalpol may be mediated by the inhibition of inflammatory mediator production in peripheral tissues.

## 5 Conclusion

In summary, the current investigation reveals that catalpol can relieve inflammatory pain caused by CFA. The basic molecular process includes the modulation of the HDAC4/PPAR-γsignaling pathway in the spinal cord of CFA rats and the stimulation of the NF-κB/NLRP3 inflammatory axis. *In vivo* and *in vitro* trials declared that the activity of spinal astrocytes and the production of inflammatory factors are also involved in the role of catalpol. In addition, catalpol alleviated peripheral pain by inhibiting tissue inflammation. In short, catalpol alleviated inflammation pain in CFA rats by targeting spinal cord and peripheral inflammation.

## Data Availability

The original contributions presented in the study are included in the article/Supplementary Material, further inquiries can be directed to the corresponding authors.
