# Supplementary figures and images for: Catalpol ameliorates CFA-induced inflammatory pain by targeting spinal cord and peripheral inflammation
Source: Front Pharmacol. 2022 Oct 24;13:1010483. doi: 10.3389/fphar.2022.1010483 (PMC9637921; doi:10.3389/fphar.2022.1010483)

FIGURE 2

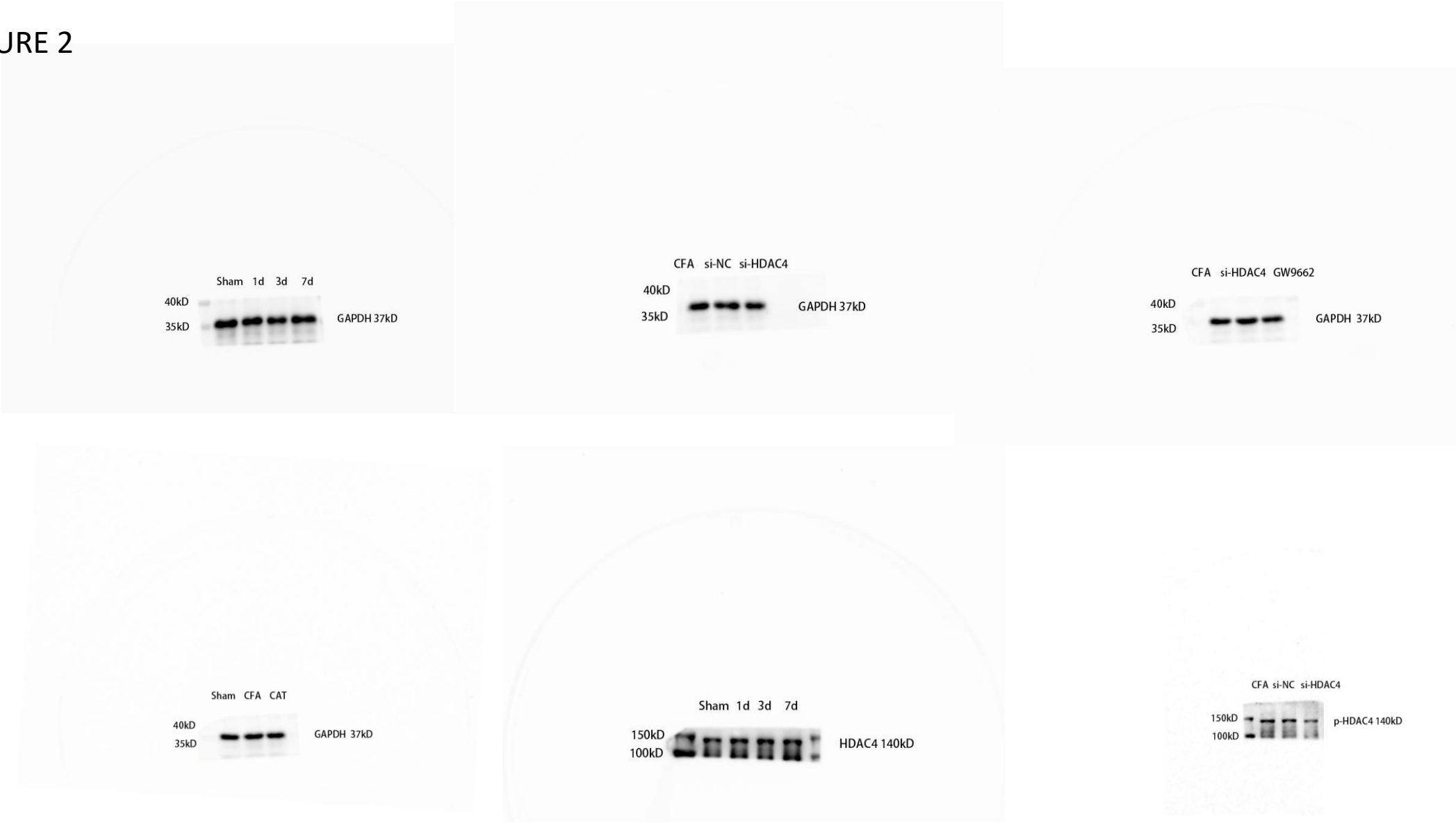

FIGURE 2

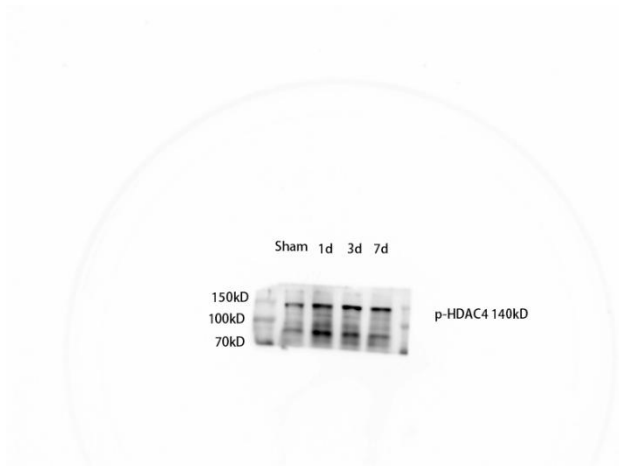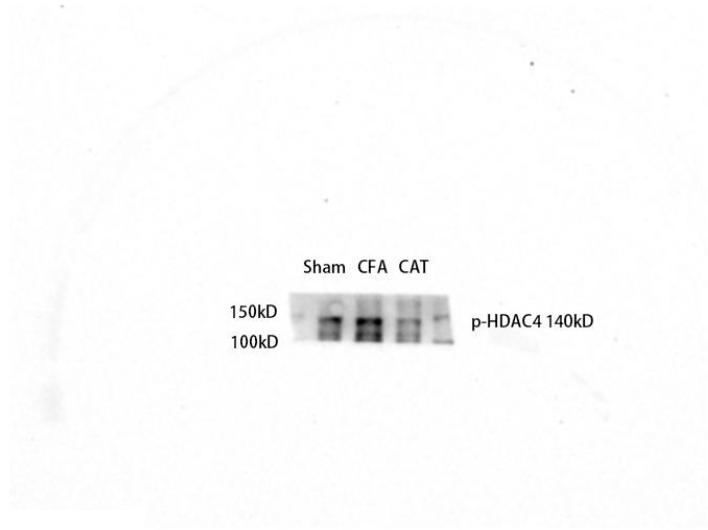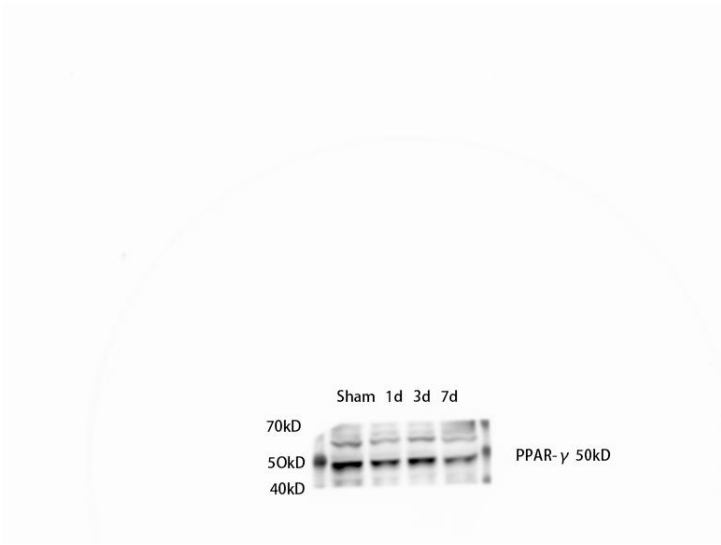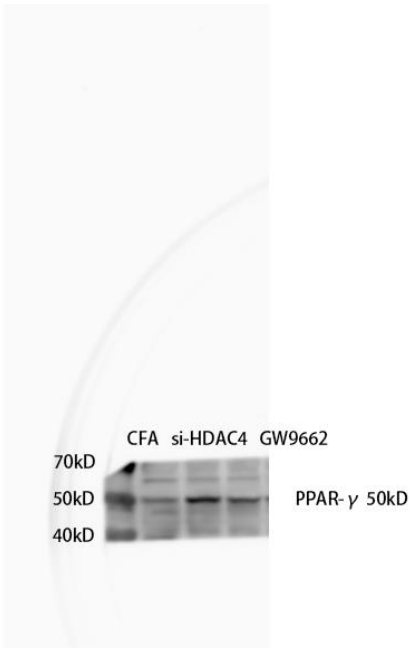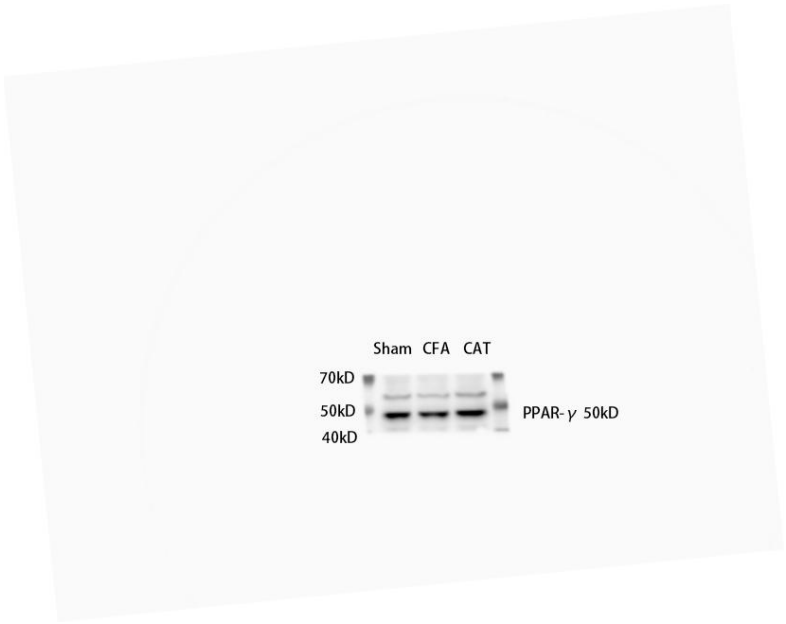

FIGURE 3

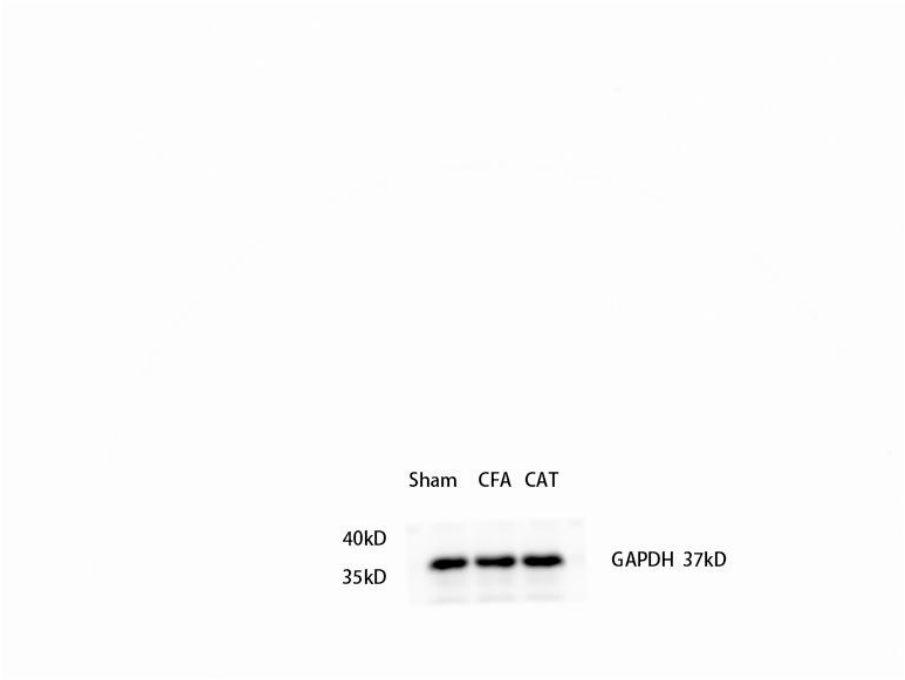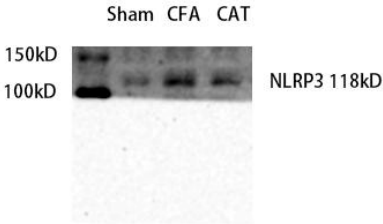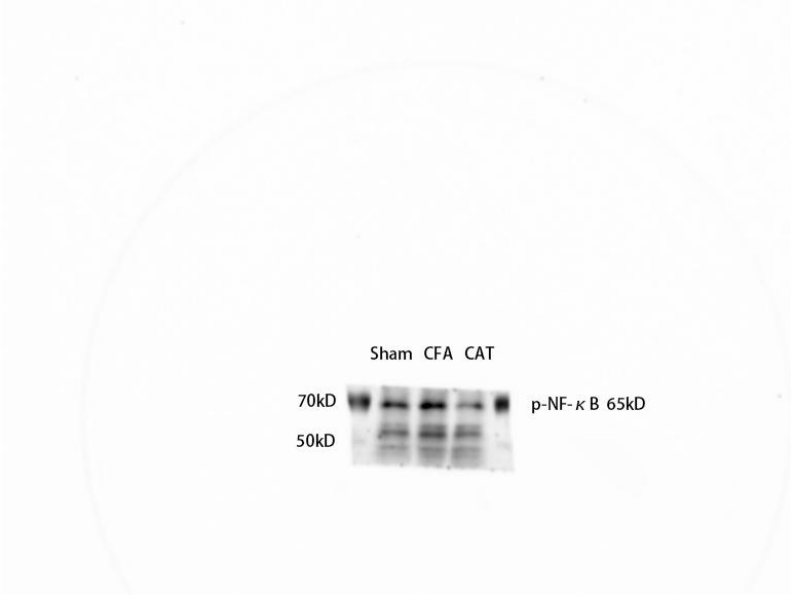

FIGURE 4

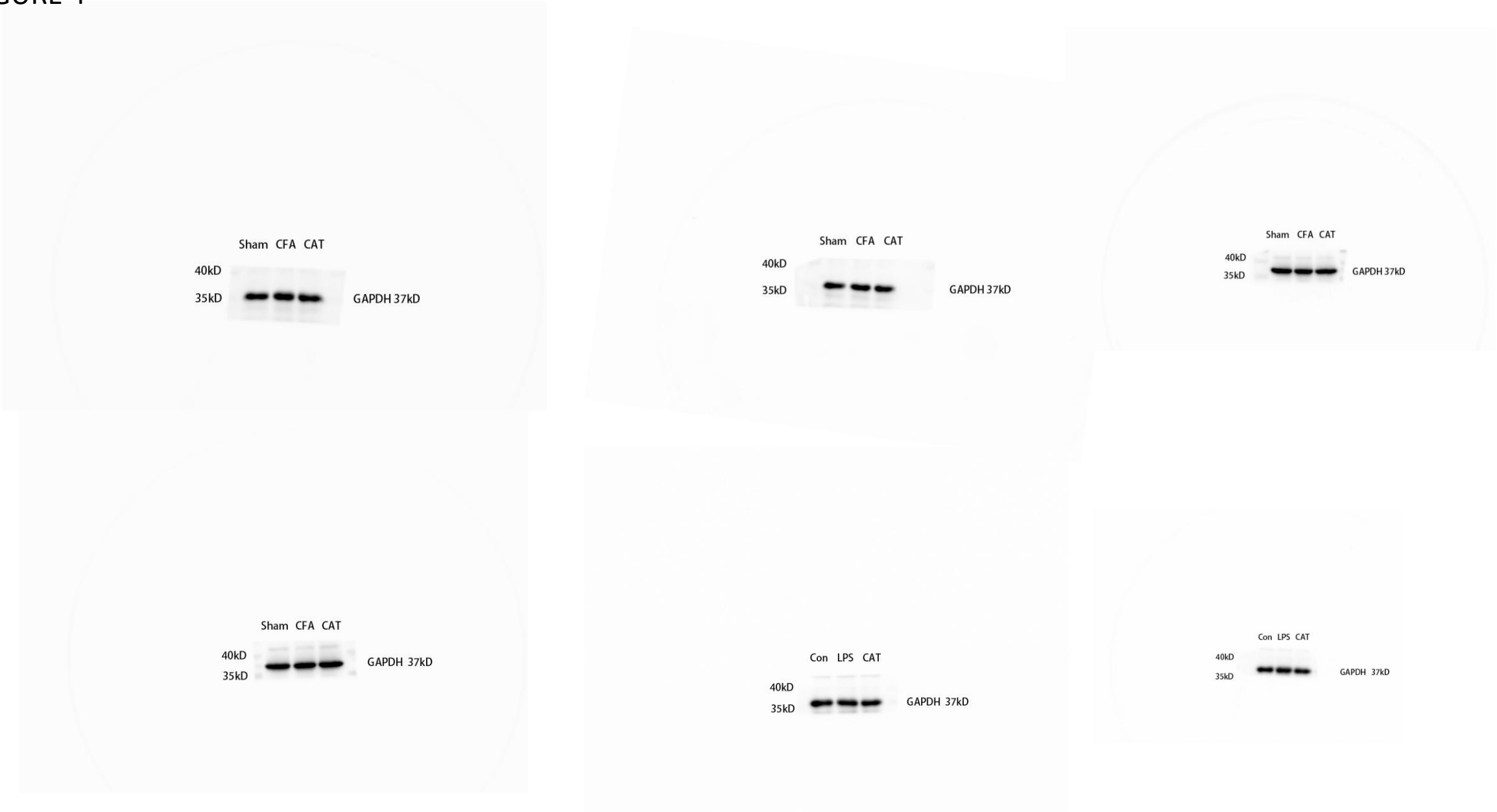

FIGURE 4

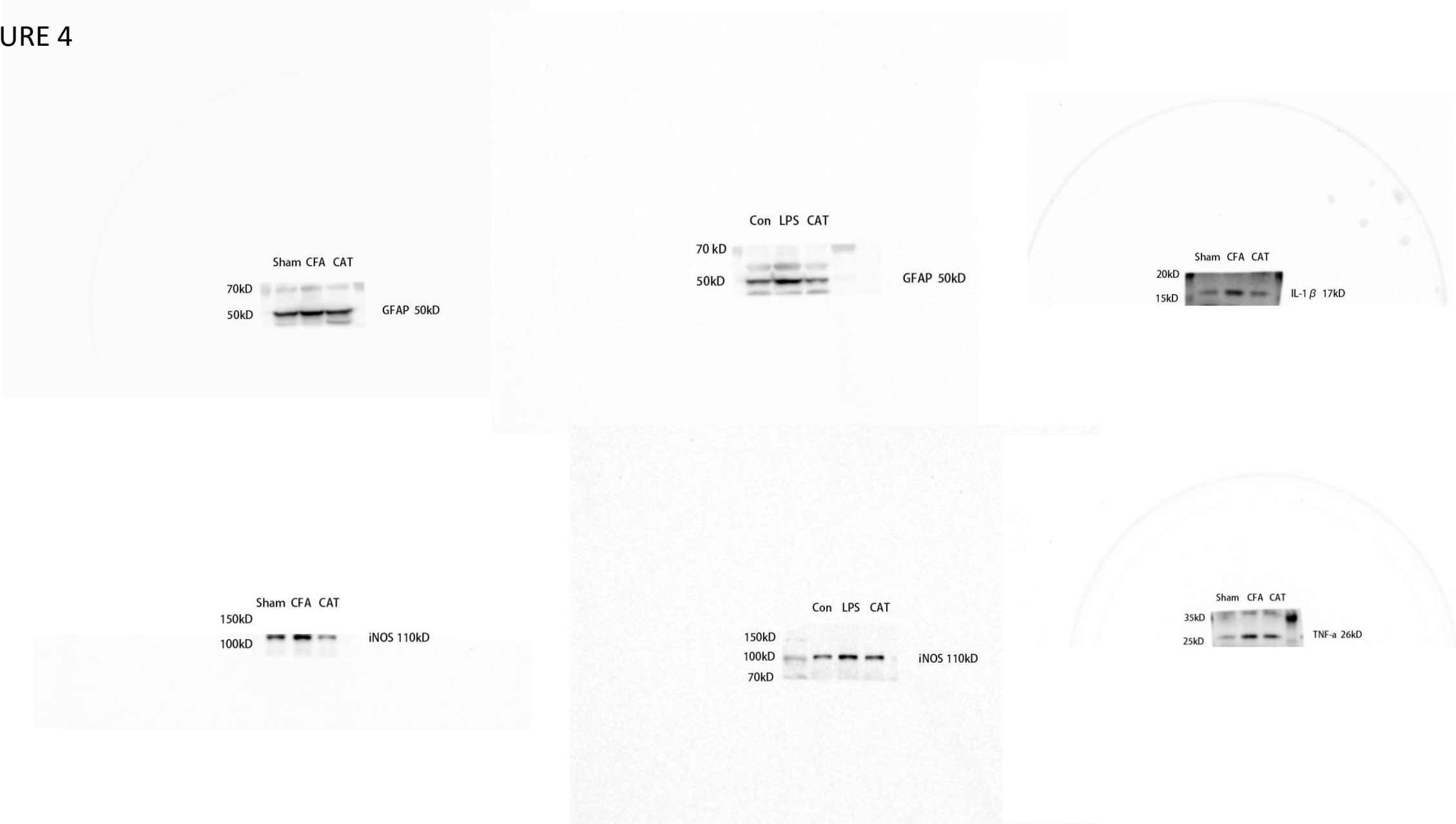

FIGURE 5

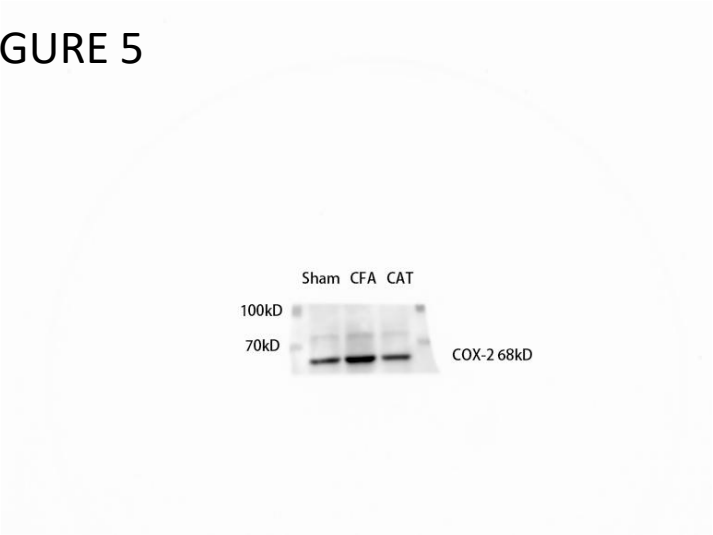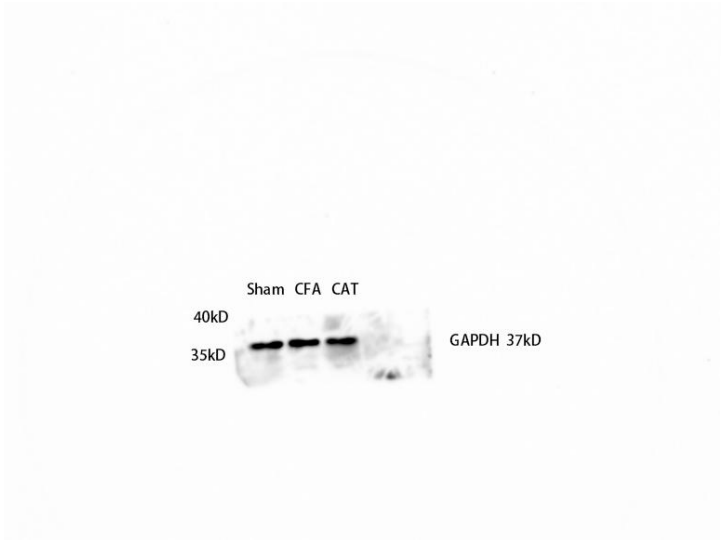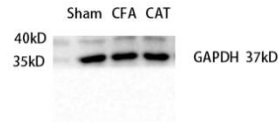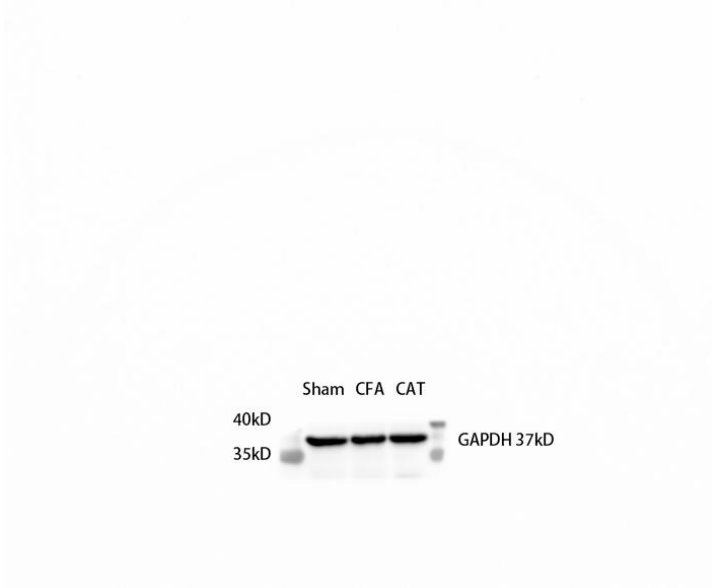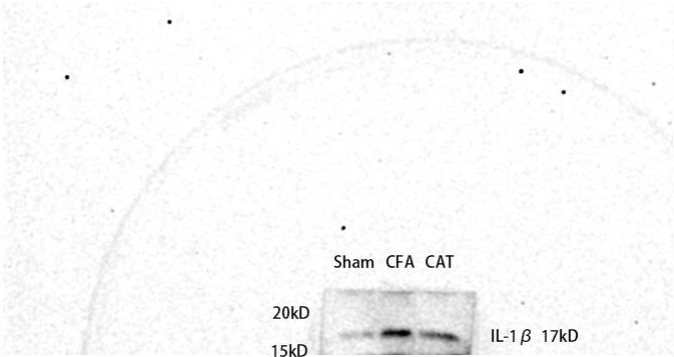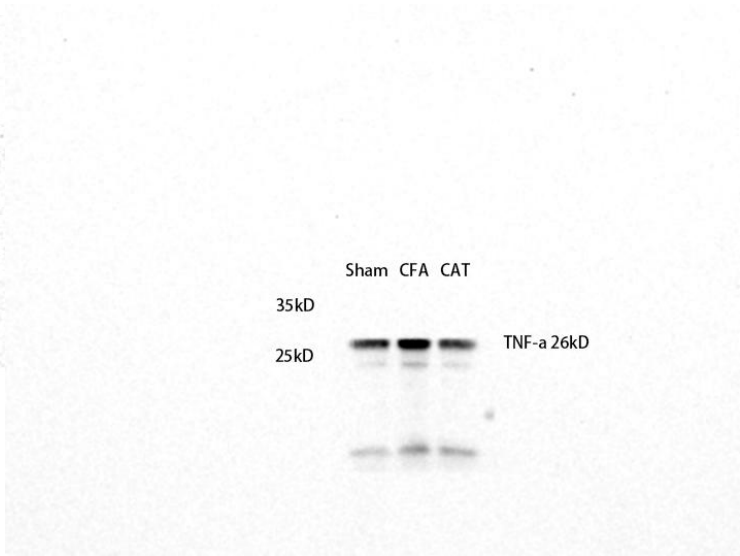

Supplement: Supplementary file 1 [file DataSheet2.PDF]

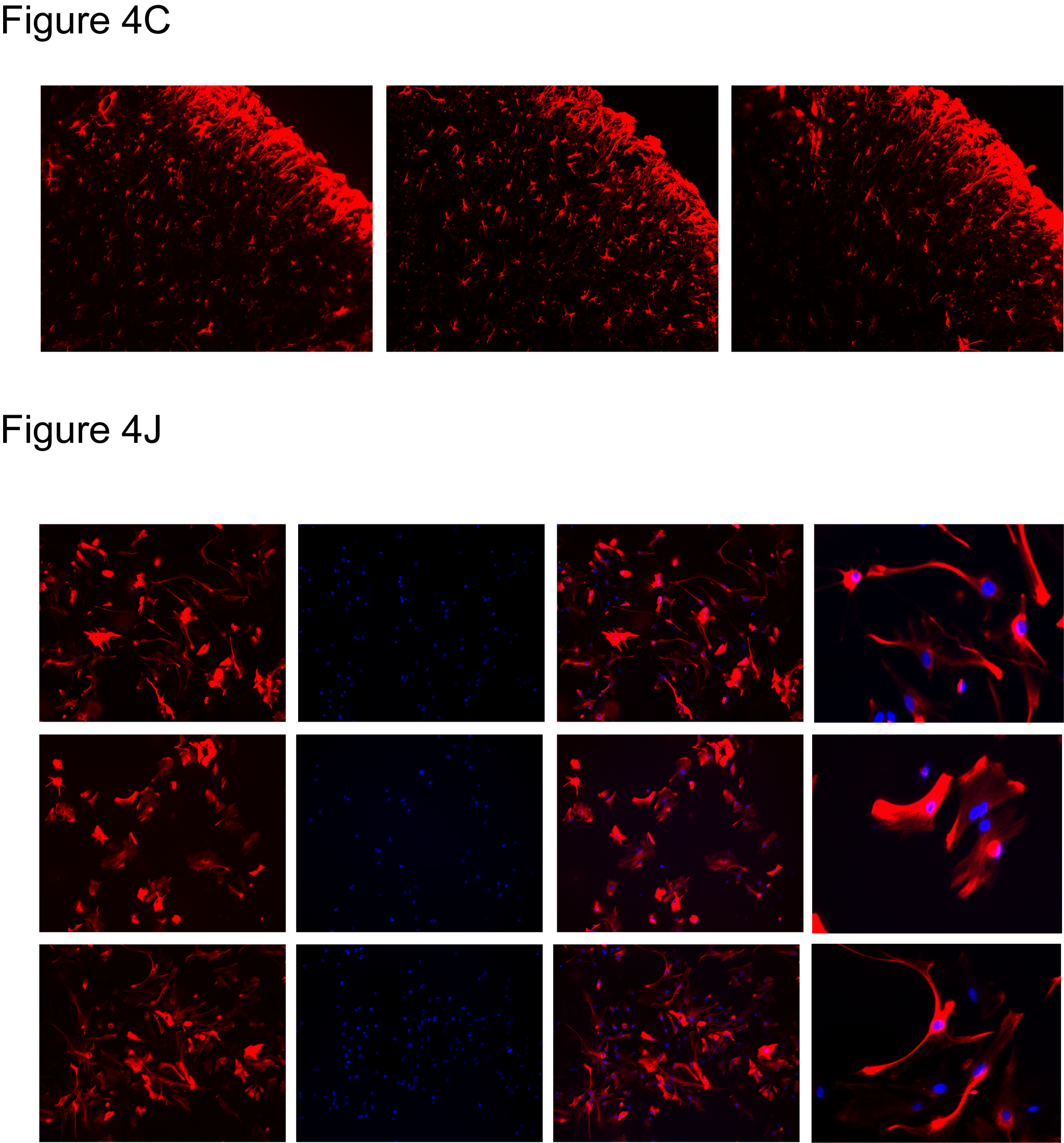

Supplement: Supplementary file 2 [file Image3.TIF]

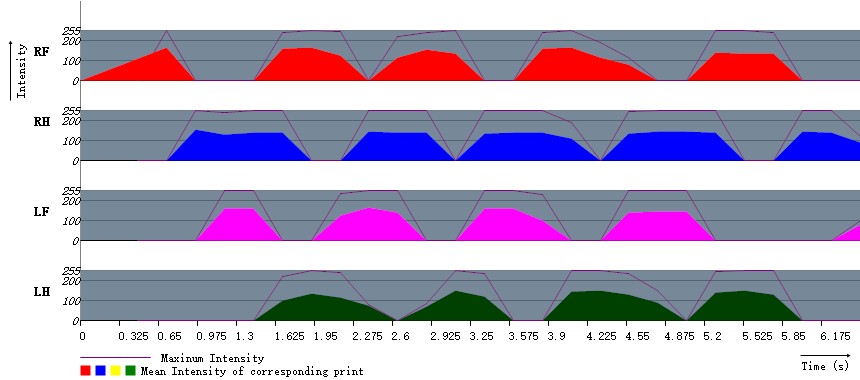

Supplement: Supplementary file 3 [file DataSheet1.ZIP › original data/CATWALK-Figure 1/CAT/Print intensity.tif]

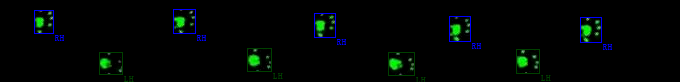

Supplement: Supplementary file 3 [file DataSheet1.ZIP › original data/CATWALK-Figure 1/CAT/Print view.tif]

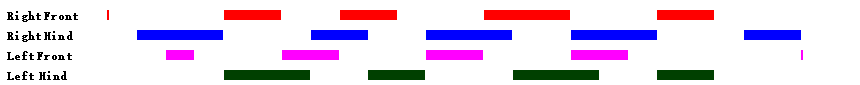

Supplement: Supplementary file 3 [file DataSheet1.ZIP › original data/CATWALK-Figure 1/CAT/timingView.tif]

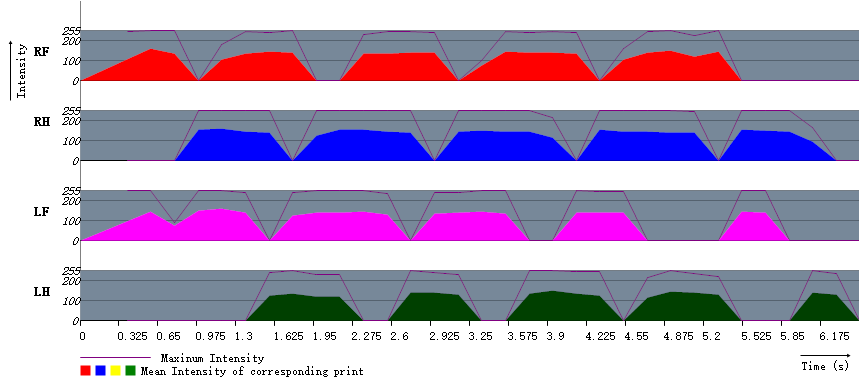

Supplement: Supplementary file 3 [file DataSheet1.ZIP › original data/CATWALK-Figure 1/CFA/Print intensity.tif]

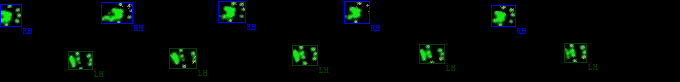

Supplement: Supplementary file 3 [file DataSheet1.ZIP › original data/CATWALK-Figure 1/CFA/Print view.tif]

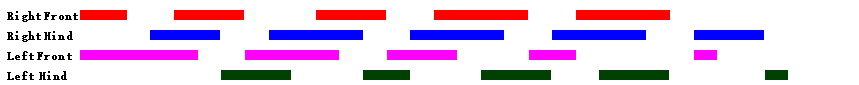

Supplement: Supplementary file 3 [file DataSheet1.ZIP › original data/CATWALK-Figure 1/CFA/TimingView.tif]

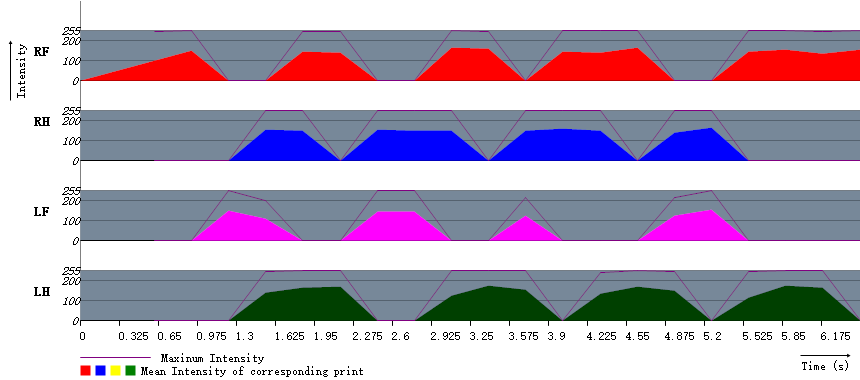

Supplement: Supplementary file 3 [file DataSheet1.ZIP › original data/CATWALK-Figure 1/Sham/Print intensity.tif]

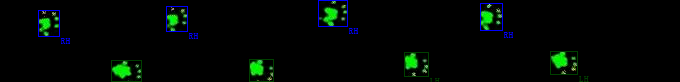

Supplement: Supplementary file 3 [file DataSheet1.ZIP › original data/CATWALK-Figure 1/Sham/Print view.tif]

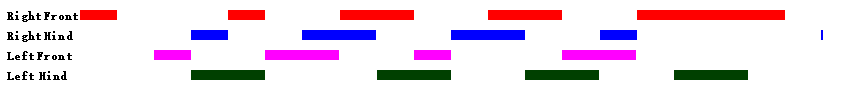

Supplement: Supplementary file 3 [file DataSheet1.ZIP › original data/CATWALK-Figure 1/Sham/TimingView.tif]

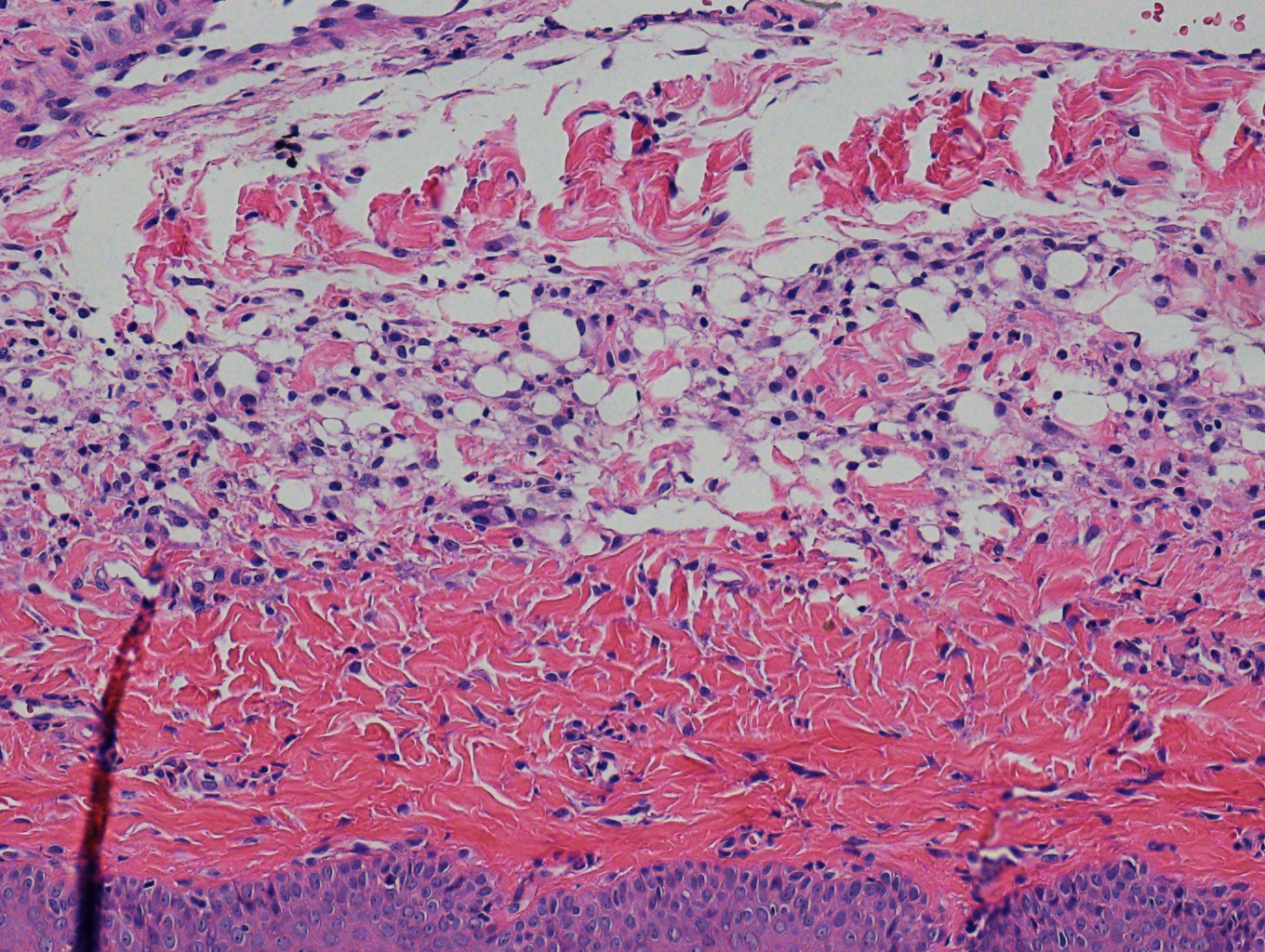

Supplement: Supplementary file 3 [file DataSheet1.ZIP › original data/H&E staining-Figure 5D/CAT.tif]

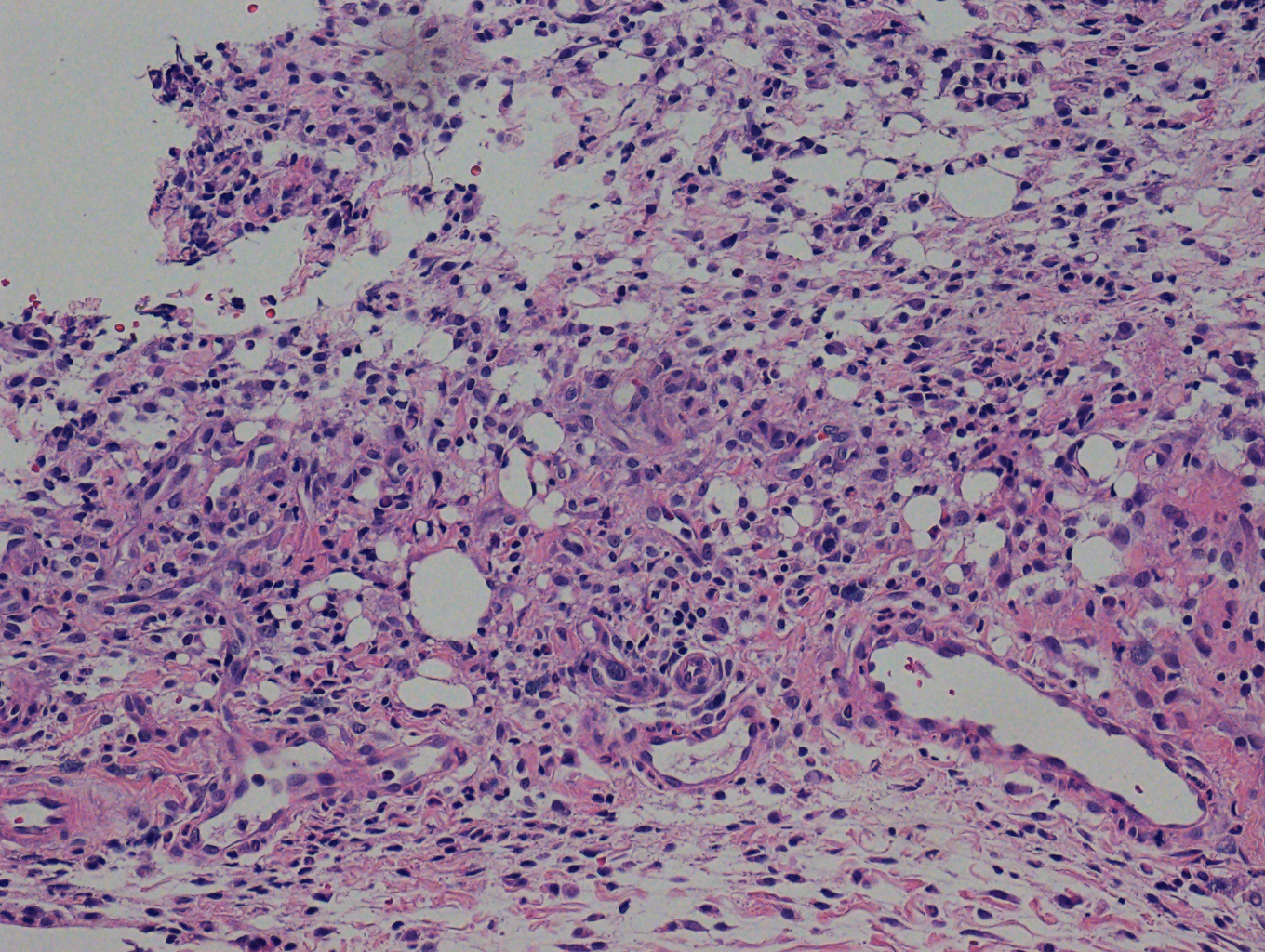

Supplement: Supplementary file 3 [file DataSheet1.ZIP › original data/H&E staining-Figure 5D/CFA.tif]

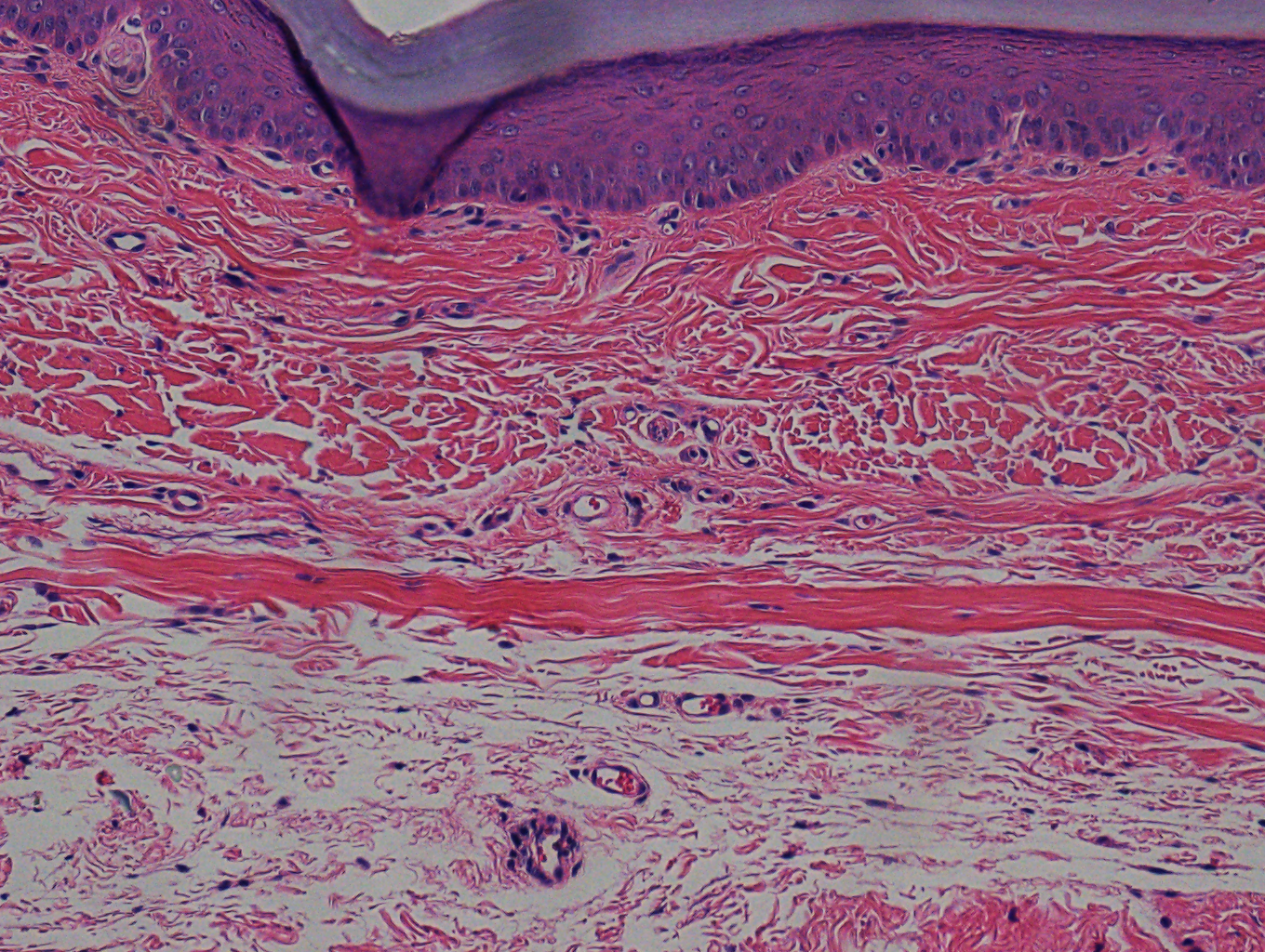

Supplement: Supplementary file 3 [file DataSheet1.ZIP › original data/H&E staining-Figure 5D/Sham.tif]

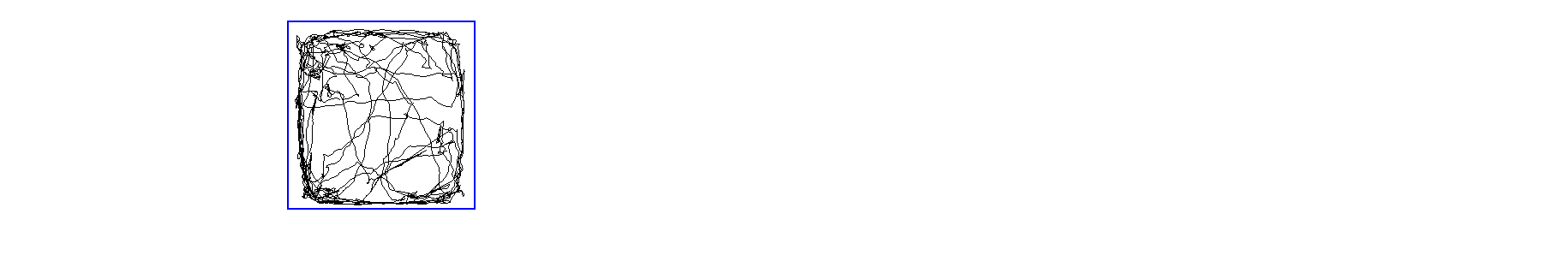

Supplement: Supplementary file 3 [file DataSheet1.ZIP › original data/the Open field-Figure 1/CAT.bmp]

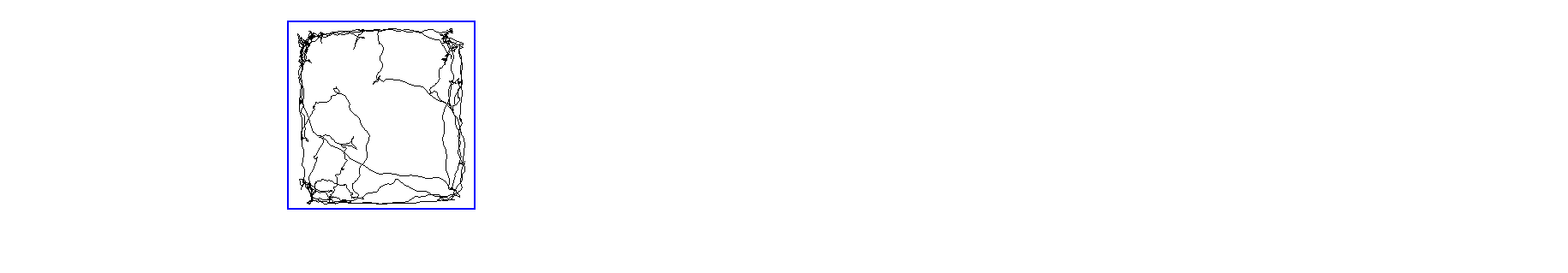

Supplement: Supplementary file 3 [file DataSheet1.ZIP › original data/the Open field-Figure 1/CFA.bmp]

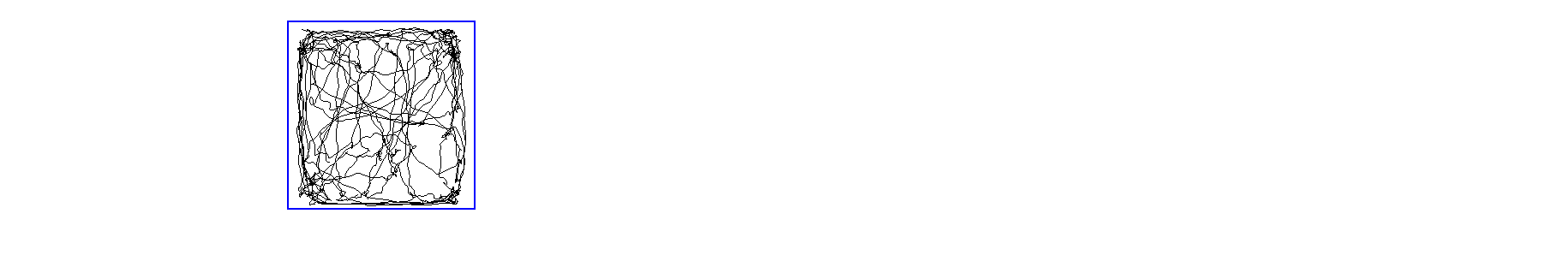

Supplement: Supplementary file 3 [file DataSheet1.ZIP › original data/the Open field-Figure 1/Sham.bmp]

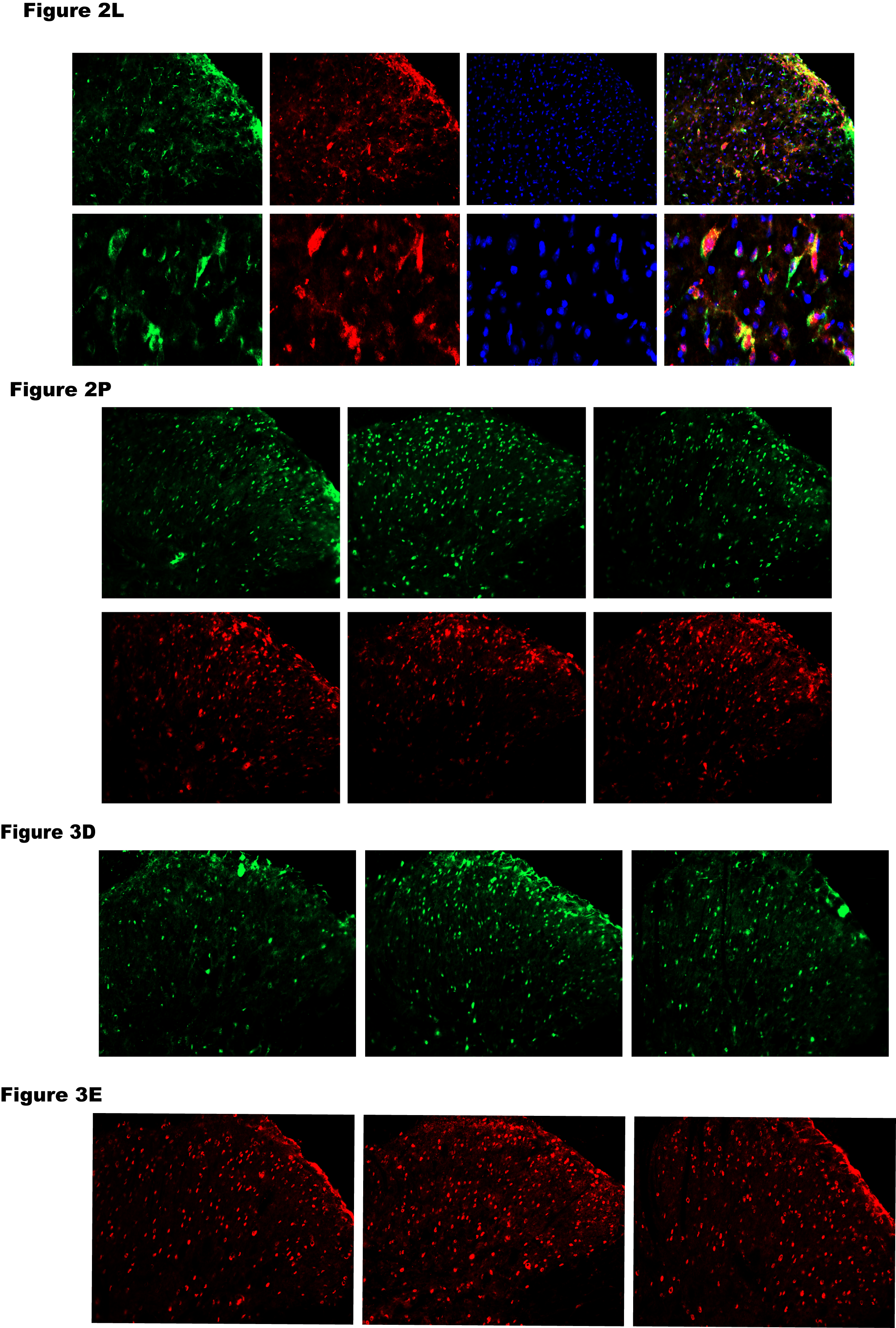

Supplement: Supplementary file 4 [file Image2.TIF]

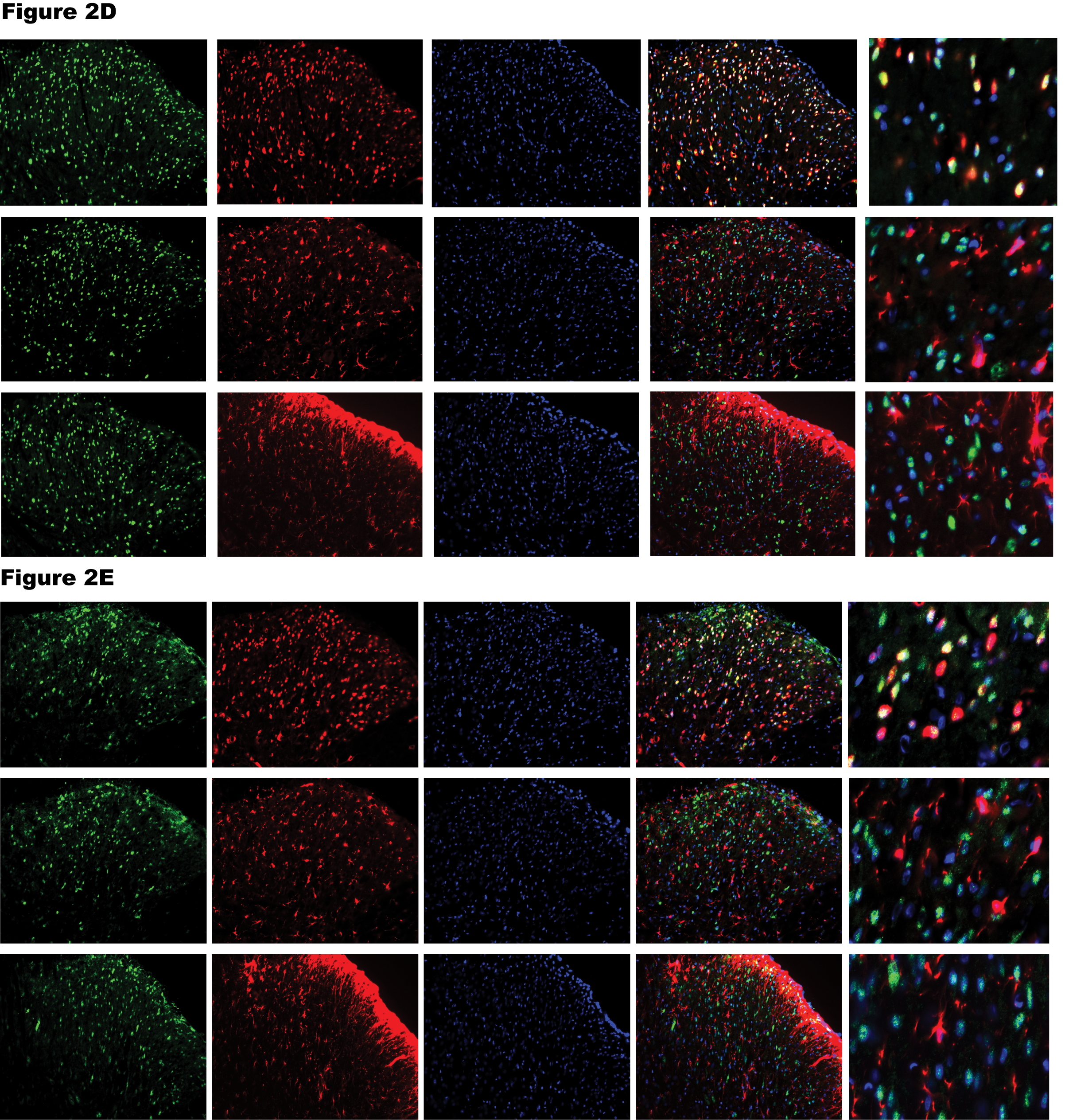

Supplement: Supplementary file 5 [file Image1.TIF]
